# Non-human primates as indicators of Kinetoplastida diversity in an urban environment in Midwest Brazil

**DOI:** 10.3389/fpara.2025.1547701

**Published:** 2025-02-17

**Authors:** Oscar Fernandes Júnior, Ana Maria Jansen, Gabriel Carvalho de Macedo, Wesley Arruda Gimenes Nantes, Filipe Martins Santos, Nayara Yoshie Sano, Wanessa Teixeira Gomes Barreto, William Oliveira de Assis, Sany Caroline Liberal, Samanta Cristina das Chagas Xavier, Fernanda Moreira Alves, Maria Augusta Dario, Carina Elisei de Oliveira, André Luiz Rodrigues Roque, Heitor Miraglia Herrera

**Affiliations:** ^1^ Programa de Pós-graduação em Ciências Ambientais e Sustentabilidade Agropecuária, Dom Bosco Catholic University, Campo Grande, Mato Grosso do Sul, Brazil; ^2^ Programa de Pós-graduação em Microbiologia, State University of Rio de Janeiro, Rio de Janeiro, Brazil; ^3^ Laboratório de Biologia de Tripanossomatídeos, Instituto Oswaldo Cruz, Rio de Janeiro, Rio de Janeiro, Brazil; ^4^ Programa de Pós-graduação em Ecologia e Conservação, Federal University of Mato Grosso do Sul, Campo Grande, Mato Grosso do Sul, Brazil; ^5^ Programa de Pós-graduação em Biotecnologia, Dom Bosco Catholic University, Campo Grande, Mato Grosso do Sul, Brazil

**Keywords:** trypanosomatids, reservoir system, urban fragments, *Sapajus cay*, *Alouatta caraya*

## Abstract

**Introduction:**

Trypanosomatids are parasites widely distributed in nature, parasitizing several host species in single or co-infections. Campo Grande (CG), capital of Mato Grosso do Sul State, is characterized by several green areas and forest fragments where wild mammals have been reported infected by diverse trypanosomatid species. In this study, we evaluated the parasitism by trypanosomatids in the non-human primates (NHP) Sapajus cay and *Alouatta caraya* sampled in three different areas of CG.

**Material and methods:**

For the detection of infections and identification of trypanosomatid species, we made hemoculture, blood smears, molecular and serological tests.

**Results:**

We detected trypanosomatids in 37/55 (67.3%) of sampled animals, all by the molecular test. DNA sequencing analyzes were performed on 32 samples, resulting in the following species identification: *Trypanosoma cruzi*, *T. minasense*, *T. rangeli*, *Leishmania (L.)* infantum and *L. (L.) amazonensis* (species already recorded in primates in Latin America), and for the first time *T. lainsoni*, a parasite related to small mammals, and *Trypanosoma* sp. DID, originally reported in marsupials *Didelphis* sp.

**Discussion:**

The detection of trypanosomatids of public health importance as *L. infantum*, *L. amazonensis* and *T. cruzi* (genotypes TcI, TcII/TcVI and TcIV) indicates the enzootic character of these species in the studied area. Also, the presence of *T. cruzi* TcIV and *T. minasense* in the conservation area supports previous studies that these parasites would be associated with the arboreal stratum. We conclude that (i) the NHP at CG participate in a complex reservoir system for parasites of great importance for Public Health in the studied area, such as *L. infantum*, *L. amazonensis* and *T. cruzi*, and (ii) there is a great diversity of trypanosomatids circulating in the urban area of this city located in the Brazilian Midwest.

## 1 Introduction

Non-human primates (NHP) coexist with humans and their domestic animals in fragmented urban areas, and this proximity over time may result in stress factors that may negatively impact their populations (de-Almeida-Rocha et al., 2017; McLennan et al., 2017; Back et al., 2019). Additionally, with 60% of free-living primates currently endangered (Estrada et al., 2017), urban forest remnants offer refuge in environments increasingly degraded, but may also pose a risk to these species due to stressor factors, including parasite transmission from humans and their domestic animals (Cibot et al., 2015; Kane and Smith, 2020; Dian et al., 2022; Lopes et al., 2022). In this sense, it is increasingly important to understand the different impacts of human encroachment that threaten primate populations.

Among the most common primate species in urban forest fragments are *Sapajus cay* and *Alouatta caraya*. Both species have a broad geographic distribution: (i) *S. cay* is distributed across Argentina, Bolivia, Paraguay and the Brazilian Midwest, and is a generalist species that has an omnivorous habit, feeding more frequently fruits and insects, eventually vertebrates (Alfaro et al., 2012; Rímoli et al., 2012; Júnior et al., 2020; Falótico, 2023); and (ii) *A. caraya*, that has a wide geographical distribution in South America from Argentina, Bolivia, Paraguay to the entire Cerrado biome in Brazil in sympatry with *S. cay*, with a folivore-frugivore diet, consisting of leaves, flowers and buds (Do Nascimento et al., 2007; Rímoli et al., 2012; Chaves et al., 2021).

Trypanosomatids (Euglenozoa; Kinetoplastea) comprise a group of ubiquitous multi-host parasites widely distributed in nature. The group is composed of flagellate protozoans with monoxenous and heteroxenous life cycles, parasitizing invertebrates, plants, or vertebrates. In general, these parasites establish long-lasting infections in mammals (Hoare, 1972; Stevens, 2008), although the epidemiological role of a given mammalian host species in the trypanosomatid transmission cycles may change according to local fauna community (Roque and Jansen, 2014). Among trypanosomatids, *Trypanosoma cruzi* and *Leishmania* spp. cause important tropical human diseases, respectively Chagas disease and leishmaniasis (Hoare, 1972).

Trypanosomatids in NHP that inhabit urban forest fragments in Brazil have been the object of some studies. Evidence of infection (presence of DNA) from *Leishmania* (*L.*) *infantum* (syn. *L.* (*L.*) *chagasi*) was found by Malta et al. (2010) in *Sapajus xanthosternos* and *Alouatta guariba* at the zoo of Belo Horizonte, Brazilian state of Minas Gerais. Paiz et al. (2019) recorded *L. infantum* in two species of marmosets (*Callithrix penicillata* and *Callithrix jacchus*) by serological and molecular tests, in the municipality of Campinas, Brazilian state of São Paulo. Cândido et al. (2021) detected *L. infantum*, *Leishmania* (*L.*) *amazonensis*, *Trypanosoma minasense* and *Trypanosoma rangeli* by molecular test in different species of NHP living in forest fragments near the city of Cuiabá, Brazilian state of Mato Grosso. *Leishmania* sp. has been detected in free-ranging *Sapajus nigritus*, by serological and molecular tests in an urban park at Brazilian Paraná state (Lopes et al., 2022); and Trueb et al. (2018) detected *Leishmania* spp. in free-living *Callithrix* sp. at urban rainforest fragments in the city of Salvador, coastal area of Brazilian Northeastern.

In Campo Grande (CG), capital of Mato Grosso do Sul (MS) state, Midwest Brazil, *S. cay* and *A. caraya* are found inhabiting urban forest fragments. This city is considered an area of intense transmission for visceral leishmaniasis due to high prevalence in humans, dogs and their vector, *Lutzomyia longipalpis* (Botelho and Natal, 2009; Brazuna et al., 2012; Cunha et al., 2014; Neitzke-Abreu et al., 2022). Furthermore, it has been reported that some wild and domestic animals including opossum *Didelphis albiventris* (Humberg et al., 2012; Nantes et al., 2021), non-hematophagous bats (de Rezende et al., 2017; Castro et al., 2020; da Silva et al., 2024; Torres et al., 2024), the carnivorous coati *Nasua nasua* (de Macedo et al., 2023), the rattlesnake *Crotalus durissus* (Nantes et al., 2024), and domestic cat (Metzdorf et al., 2017) compose the reservoir system for *L. infantum* in CG city. The causative agent of the American Cutaneous Leishmaniasis, *L. amazonensis*, have been reported to circulate in CG, being detected in phyllostomid bats, *D. albiventris*, and *N. nasua* [27.31, de Macedo GC, unpublished data], as well as in *Bichromomyia flaviscutellata*, its main vector (Neitzke-Abreu et al., 2022). Furthermore, regarding *Trypanosoma* species found parasitizing vertebrates in CG, Nantes et al. (2021) showed *T. cruzi* DTU TcII, *Trypanosoma lainsoni* and an unidentified *Trypanosoma* spp. DID in *D. albiventris*; Torres et al. (2024) isolated *T. cruzi* DTU TcI, *T. cruzi marinkellei*, *Trypanosoma dionisii*, and *Trypanosoma janseni* in non-hematophagous bats; and Nantes et al. (2024) detected *T. cruzi* DTU TcII/VI, *Trypanosoma* sp. DID, *Trypanosoma cascavelli*, and Trypanosomatidae CROT in *C. durissus* and *Bothrops moojeni*.

Because NHPs are found inhabiting urban forest fragments at CG and are described as hosts of different trypanosomatid species in South America, and diverse trypanosomatid species are described in wild hosts from CG, we aimed to investigate the richness of trypanosomatid infections in the *S. cay* and *A. caraya* at CG. Moreover, we discuss the role of these PNH species in the maintenance of *T. cruzi* and *Leishmania* spp., parasites of public health importance.

## 2 Materials and methods

### 2.1 Study areas and captured animals

**Figure 1:**
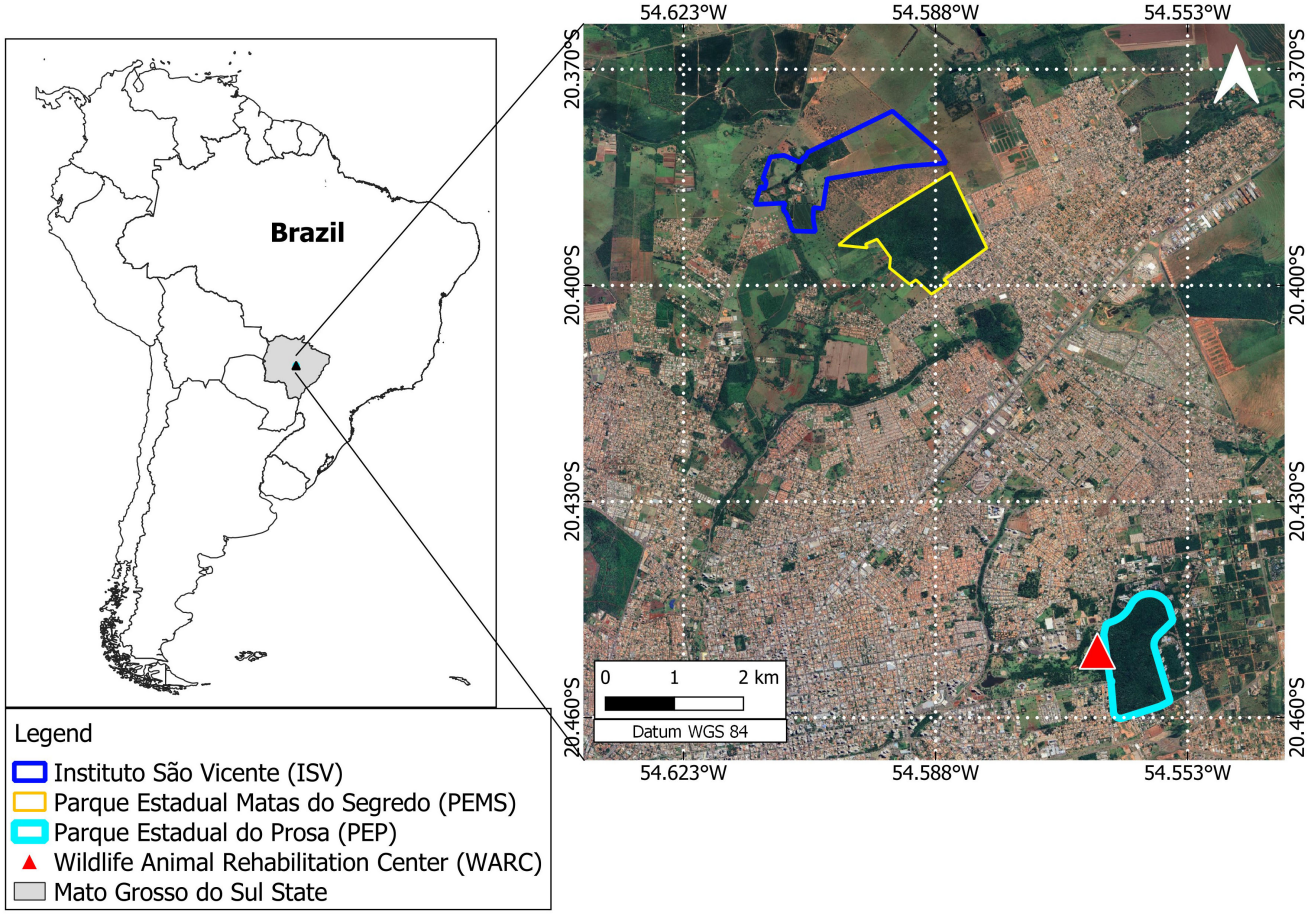
Studied area belonging to the Cerrado Biome. In detail are the anthropized area “Instituto São Vicente” (ISV), the preserved conservation unit “Parque Estadual Matas do Segredo” (PEMS) and the Wildlife Rehabilitation Center (WRC) located within the conservation unit “Parque Estadual do Prosa” (PEP).

The Capuchin monkeys (*S. cay*) were captured in two forested fragments from Campo Grande (CG) municipality, Mato Grosso do Sul state, Midwest Brazil: “Instituto São Vicente” (ISV) and “Parque Estadual Matas do Segredo” (PEMS). Approximately 2.15 km apart (**Figure 1**), both areas present phytophysiognomy of the savannah forest, riparian forest and lowland areas. The ISV is an anthropized complex of 191 hectares of mixed land use, divided into forested areas, mixed crops (corn, beans and fruits) and a school farm from the Dom Bosco Catholic University (UCDB) with livestock production practices. The PEMS is a preserved conservation unit covering an area of 180 hectares that harbor several wildlife species as the coati (*N. nasua*), crab-eating fox (*Cerdocyon thous*), puma (*Puma concolor*), tapir (*Tapirus terrestres*), opossum (*D. albiventris*), and gracile mouse opossum (*Gracilinanus agillis*). We also included samples from *S. cay* and Howler Monkeys (*A. caraya*) from the local Wildlife Rehabilitation Center (WRC), located within another conservation unit, the “Parque Estadual do Prosa” (PEP) (**Figure 1**). The WRC receives dozens of wildlife species (mammals, birds, reptiles) from the illegal trade, run over and voluntarily delivered by the local people. The primates sampled at the WRC were kept in captivity for a period ranging from six months to four years and derived from different municipalities in the MS.

### 2.2 Capture procedures

From September to November 2020, the capuchin monkeys were habituated to box traps (90 x 45 x 50 cm) installed on the top of two-meter platforms at ISV and PEMS, using fruits and eggs inside the open traps. Afterwards, from December 2020 to September 2021, the captures took place during five consecutive days every three months. The traps were opened at dawn (05:00 am), checked up every 30 min, and closed around 01:00 pm of the same day. The total effort was 7,680 hour-trap. At the WRC the captive animals were caught using nets.

The sedation protocol consisted of a combination of midazolam hydrochloride (0.5 mg/kg) and ketamine hydrochloride (12 mg/kg), and benzodiazepine flumazenil (0.025 mg/kg) (LANEXAT^®^, Roche) as a reverser. All captured animals were tagged with subcutaneous transponders (AnimallTag^®^, Brazil). After total recovery from sedation, the animals were released at the capture sites in the ISV and PEMS, and into their respective cages at the WRC. All field procedures were performed in accordance with the “Instituto Chico Mendes de Conservação da Biodiversidade” (license number 70946-3), the “Instituto de Meio Ambiente de Mato Grosso do Sul” (006/2020, process No. 71/401070/2020), and the Ethics Committee for Animal Use of UCDB (license number 001/2020).

### 2.3 Biological sample collection

Blood and bone marrow samples were collected via the femoral vein and *manubrium sterni* respectively, after an asepsis protocol containing bactericidal soap, iodine, and 70% alcohol. Blood samples were taken using 24G hypodermic needles and bone marrow samples were obtained using 18G needles. Both samples were deposited in EDTA (Ethylenediamine tetraacetic acid) tubes. Blood samples were also deposited in clot activator tubes to obtain serum and clot samples, adding absolute ethanol (1:1) to the clot samples. All samples were kept under refrigeration (−20°C) until laboratory analysis. We also performed blood smears of peripheral blood from the tip of the ear.

### 2.4 Parasitological test

Axenic cultures were performed by placing 0.3 ml of blood in biphasic media Novy-MacNeal-Nicolle (NNN) containing Liver Infusion Tryptose overlay, and 0.2 ml of bone marrow in biphasic media NNN containing Schneider’s Insect medium overlay, both supplemented with 10% fetal bovine serum. The tubes were incubated at 26–28°C and examined weekly (bone marrow cultures) or every two weeks (hemocultures) for up to two and four months, respectively. The positive axenic culture at exponential phase was amplified and deposited in the COLTRYP-FIOCUZ/RJ.

Blood smears were stained using Panoptic kit (Laborclin^®^, Brazil) and examined for trypanosomatid flagellates under optical microscopy at 400x and 1000x magnifications. Positivity in blood cultures and/or blood smears was considered indicative of high parasitemia and potential to serve as a source of infection for vectors at the time of capture.

### 2.5 Serological diagnosis

A serological survey for the detection of anti-*Leishmania* and anti-*T. cruzi* IgG antibodies was performed using an Indirect Immunofluorescent Antibody Test (IFAT) as previously described by Camargo (1966). The antigens used in *Leishmania* spp. serology were an equal mixture of the strains MHOM/BR/1975/M2903 (*Leishmania braziliensis*-IOC/L566) and MHOM/BR/1974/PP75 (*L. infantum*-IOC/L579) obtained from the Collection of *Leishmania* from Oswaldo Cruz Institute (CLIOC-FIOCRUZ/RJ) from the Oswaldo Cruz Foundation (FIOCRUZ – Rio de Janeiro – RJ/Brazil), and in serology to *T. cruzi* we used an equal mixture of parasites derived from the strains M000/BR/1974/F90 (TcI) and MHOM/BR/1950/Y (TcII), obtained from the Collection of *Trypanosoma* from Wild, Domestic and Vector Mammals (COLTRYP-FIOCUZ/RJ) from FIOCRUZ. Sera from all primates were tested with anti-monkey IgG conjugated with Fluorescein Isothiocyanate (Sigma, St. Louis, MO, USA), and the cut-off point adopted was 1:10 (Lisboa et al., 2004). In addition to IFAT, the sera were submitted to ELISA (Enzyme-Linked Immunosorbent Assay) (ELISA, Biomanguinhos, Rio de Janeiro, Brazil) using anti-monkey peroxidase-conjugated antibodies (IgG) and the same antigens used for IFAT. The cut-off point was established by the mean OD (Optical Density) of the negative control ± three standard deviation and the gray range adopted was 20% above the cut-off value (Kerr et al., 2016). For each serological assay, two positive and two negative controls for *Leishmania* spp. and *T. cruzi* were added. We adopted as seropositivity criteria the positive reaction in both serological tests (IFAT and ELISA).

### 2.6 Molecular tests

The obtained parasite culture was subjected to DNA extraction using the phenol-chloroform method (Michael and Joseph, 2012). Genomic DNA of clots was extracted using the ammonium acetate method (Rodrigues et al., 2019), while the DNA of blood and bone marrow samples was extracted using the QIAamp Blood DNA Mini Kit (Qiagen^®^, Germany) according to the manufacturer instructions. Total DNA was diluted with 20 μl of elution buffer and stored at −20°C until molecular diagnosis. Detection of trypanosomatids in clots, blood, and bone marrow, besides the axenic culture, was performed by the Nested Polymerase Chain Reaction (nPCR) targeting the trypanosomatid 18S rDNA gene (~650 bp) (Smith et al., 2008). The DNA extracted from the bone marrow samples was also subjected to a conventional PCR targeting the conserved region of the kinetoplast (kDNA) (120 bp) for *Leishmania* spp. detection (Schubach et al., 1998).

The products of nPCR and from the axenic culture were purified using the Illustra GFX PCR DNA and gel band purification kit (GE Healthcare Life Sciences, Little Chalfont, Buckinghamshire, UK). The axenic culture sample was sequenced for both strands of DNA with BigDye Terminator v3.1 Cycle Sequencing Kit (Applied Biosystems, Foster City, CA, USA) on an ABI 3730 DNA sequence available on the PDTIS/FIOCRUZ sequencing platform. The sequences were edited, aligned, and corrected using BioEdit software.

DNA from 18S rDNA nPCR positive samples was quantified using the broad-range Qubit DNA fluorimeter (Invitrogen^®^, United States). Samples with a minimum concentration of 20 ng/µl in a volume of 20 µl, totalizing a minimum of 400 ng per sample of DNA, were subjected to amplicon Next Generation Sequencing (NGS) according to Illumina recommended protocols (Illumina Demonstrated Protocol: Metagenomic Sequencing Library Preparation) and sequenced on an Illumina HiSeq2500 (PE250) platform, using 18S rRNA primers described by Barbosa et al. (2017).

### 2.7 Statistical analysis

In order to verify if there were differences in 18S rDNA detection among different templates (blood, clot and bone marrow), we performed a Cochran Q test (x²-based Cochran Q statistic) for related samples, followed by a McNemar *Post hoc* test. Also, to test if there was a match between the models, we performed a Fleiss Kappa test. Chi-squared test (p < 0.05) was applied to verify differences in 18S rDNA detection between females and males. All statistical analyses were performed through the R software (R Core Team, 2022).

### 2.8 Bioinformatics analysis

The NGS-generated data were imported in the R v3.6.2 platform in which all the analysis was carried out (R Core Team, 2022). Sequences were analyzed using DADA2 package v1.14.0 following the analysis pipeline as given in the tutorial (https://benjjneb.github.io/dada2/tutorial.html) (Callahan et al., 2016). Further, taxonomy was assigned using SILVA v132. The Amplicon Sequence Variant (ASV) table, assigned taxonomy, and sample metadata information were combined as a phyloseq object (phyloseq package version 1.30.0) (McMurdie and Holmes, 2013). As a read cut-off for determining the species occurrence per sample, the total reads per sample obtained in the ASV table were normalized to 100,000 reads and AVS that presented ≤ 50 reads in the sample were excluded from the analysis according to Dario et al. (2022a).

### 2.9 Phylogenetic analysis

The ASV reads were aligned to other kinetoplastid 18S rRNA sequences retrieved from the GenBank (https://www.ncbi.nlm.nih.gov/genbank/) database using the algorithm L-INS-i in MAFFT v.7.0 software (Katoh and Standley, 2013).The alignment was inspected and manually edited on Mega7 (Kumar et al., 2016). Maximum likelihood (ML) and Bayesian inference (BI) were performed. For each analysis, the best base substitution models were chosen according to the corrected Akaike information criterion (cAIC) in jModelTest-2.1.10 (Darriba et al., 2012). ML reconstruction was performed in the IQ-Tree software (Nguyen et al., 2015) with branch support of 5000 replicates with 1000 maximum interactions and 0.99 minimum correlation coefficients by ultrafast bootstrapping (Hoang et al., 2018). The SH-aLRT branch test was performed with 5000 replicates to validate the ultrafast bootstrapping result. The heuristic search method used was the program’s default and the algorithm to obtain the final tree was Neighbor joining (NJ). All these analyses were available on PhyloSuite v.1.2.2 software (Zhang et al., 2020).

Bayesian inference was performed in Bayesian Evolutionary Analysis Sampling Trees (BEAST) v2.6.2 (Bouckaert et al., 2019) using the Bayesian Markov chain Monte Carlo (MCMC) method to assign kinetoplastid prior to information. The Yule model specification was used for species tree reconstruction. Seven independent runs were performed for 20M with sampling every 2000 generations and pre-burn-in of 5M. The runs converged and the effective sample size (ESS) were observed in TRACER v.1.6 (Rambaut et al., 2018). Parameters greater than 500 were considered appropriate. The final tree was generated with maximum clade credibility (MCC) based on 15002 trees (burn-in = 5000) and a 0.6 posterior probability limit (PP) in Tree Annotator. The ML and BI reconstruction trees were visualized in Figtree v.1.4.3.

## 3 Results

A total of 50 NHP were sampled: 11 capuchin monkeys in ISV (five females and six males), and 27 in PEMS (15 females and 12 males). At the WRC, eight capuchin monkeys (one female and seven males) and four Howler monkeys (three females and one male) were sampled (**Table 1**). During the field works, five capuchin monkeys were recaptured: two in ISV (two females), and three in PEMS (two females and one male), totaling 55 samples.

**Table 1:** Infections by trypanosomatids in capuchin monkeys (*Sapajus cay*) and Howler monkeys (*Alouatta caraya*) sampled in Campo Grande, Midwest Brazil.

Area	Specie	ID	County	Template	TcI	TcII/TcVI	TcIV	*L. infantum*	*L. amazonensis*	*T. minasense*	*T. rangeli*	*T. lainsoni*	*Trypanosoma* sp. DID	*Bodo* sp.
Wild Rehabilitation Center (WRC)	*Alouatta caraya*	554	Campo Grande	Blood			x			x			x	
		553	Bela Vista	Blood			x		x	x			x	
		552	Campo Grande	Blood			x	x	x	x			x	
		555	Campo Grande	Blood		x	x	x		x			x	
	*Sapajus cay*	494	Sonora	Blood				x		x			x	
		493	Miranda	Blood			x			x			x	
		550	Campo Grande	Blood			x	x	x				x	
		551	Campo Grande	Blood		x	x	x		x			x	
		496	Cassilândia	Clot+Blood		x		x					x	
		548	Campo Grande	Clot+Blood		x		x					x	
		549	Jaraguari	Clot+Blood		x	x					x	x	
		495	Campo Grande	Bone marrow				x		x			x	
Anthropized Area (ISV)	*Sapajus cay*	298A	Campo Grande	Clot	x	x		x			x		x	
		297	Campo Grande	Clot	x			x					x	
		460A	Campo Grande	Clot	x	x		x					x	
		489	Campo Grande	Clot+Blood				x	x		x		x	x
Conserved Area (PEMS)	*Sapajus cay*	307	Campo Grande	Blood	x	x		x		x			x	
		457	Campo Grande	Blood	x	x		x		x			x	
		458	Campo Grande	Blood	x	x		x		x			x	
		486	Campo Grande	Blood	x	x	x	x		x			x	
		488	Campo Grande	Blood	x	x	x	x		x			x	
		490	Campo Grande	Blood		x	x	x		x			x	
		491	Campo Grande	Blood			x	x		x			x	
		492	Campo Grande	Blood			x	x		x			x	
		304B	Campo Grande	Blood									x	
		304A	Campo Grande	Clot	x	x	x						x	
		306	Campo Grande	Clot		x	x	x					x	
		480B	Campo Grande	Clot+Blood									x	
		300B	Campo Grande	Clot+Blood		x							x	
		302	Campo Grande	Clot+Blood	x	x		x	x				x	
		466	Campo Grande	Bone marrow	x	x		x					x	
		465	Campo Grande	Bone marrow	x	x		x	x	x			x	

Discrete Typing Units of *Trypanosoma cruzi*: TcI, TcII/TcVI and TcIV. The x means the presence of the parasite in the sample.

A single culture from blood was positive for *T. rangeli*, while all blood smears and bone marrow cultures were negative for trypanosomatids. Also, all *Leishmania* sp. specific PCR performed in bone marrow samples were negative. We detected trypanosomatids in 37/55 (67.3%) samples by Nested PCR for 18S rDNA gene. From these 37 positive samples, we found differences according to templates: blood (n = 18), clot (n = 6), bone marrow (n = 6) and both clot + blood (n = 7). Indeed, the Cochran Q test showed that there was significative difference among the rates of infection by templates (x²-based Cochran Q = 13.9, df = 2, p-value = 0.000939), mainly between blood and clot (x² = 6.2609, df = 1, p-value = 0.01234) and between blood and bone marrow (x² = 9.4815, df = 1, p-value = 0.002076). Also, we found no agreement in 18S rRNA detection by templates (Kappa = 0.00107, p-value = 0.989) and we did not observe differences in 18S rDNA detection between sex (x² = 1.3889, df = 1, p-value = 0.2386). Furthermore, none of the tested PNHs were serological positive for the two performed tests.

Overall, the infection rate for trypanosomatids (18S rDNA) was similar between the preserved PEMS (16/27 59.3%) and anthropized ISV (6/11 54.5%) areas. In the WRC all sampled animals (n = 12) were infected by trypanosomatids. Considering the five recaptured animals, four were positive for trypanosomatids in the first and second collection, within an interval up to nine months between captures. Only the female 480 captured in the PEMS was negative in the first collection and positive three months later.

**Figure 2:**
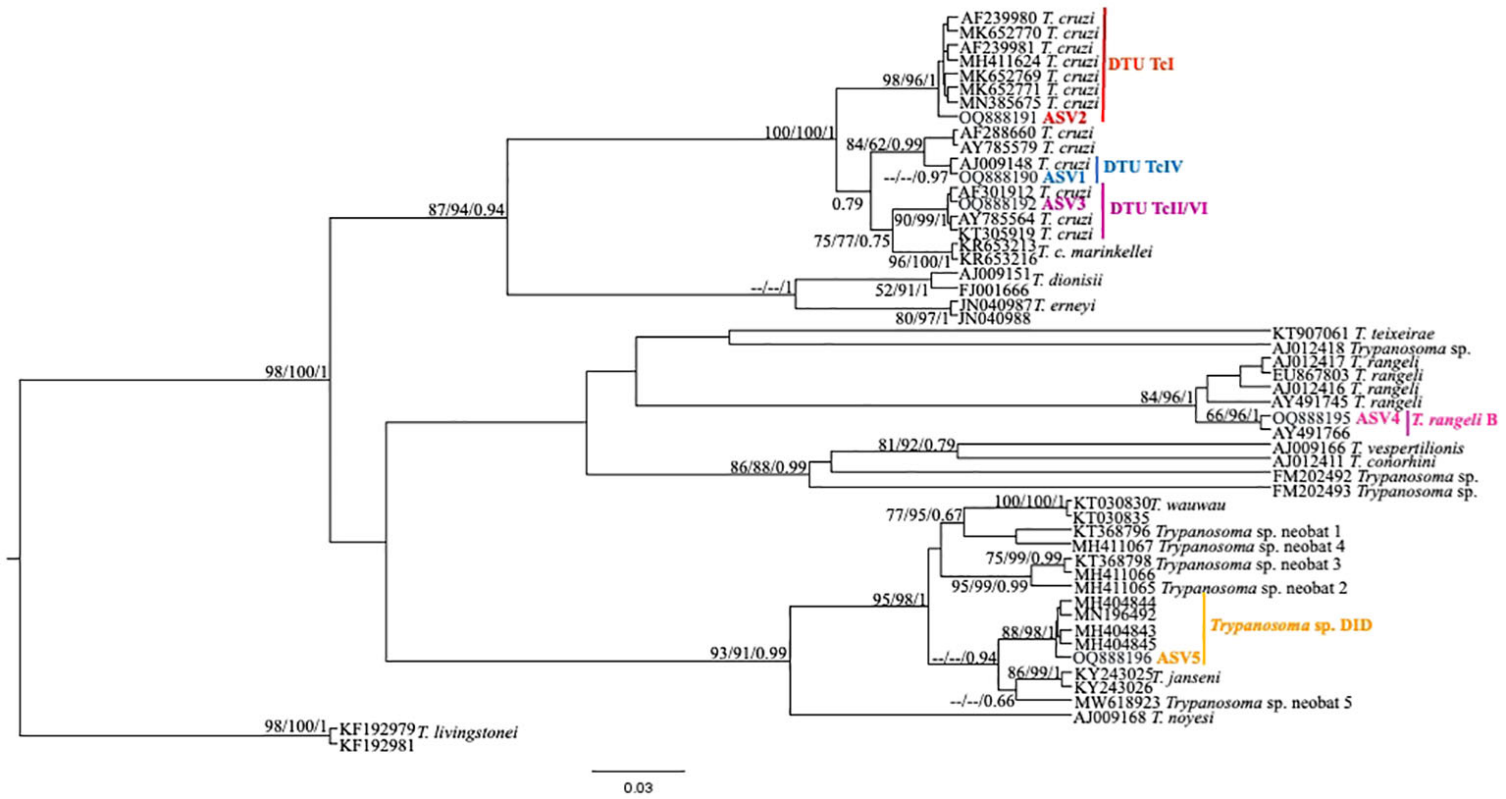
*Trypanosoma cruzi* clade phylogenetic tree based on 18S rDNA gene for whole blood DNA from different primate species. The trees were inferred with the Tamura Nei model plus gamma distribution among sites (TN+G). The number at nodes corresponds to SH-aLRT, ultrabootstrap (ML) and posterior probability (BI), respectively. The scale bar shows the number of nucleotide substitutions per site. The red line indicates the group formed by *T. cruzi* DTU TcI; the blue line indicates the sequences grouped as *T. cruzi* DTU TcIV; the purple line indicates the sequences groups as *T. cruzi* DTU TcII/VI; the pink line indicates the sequences identified as *T. rangeli* lineage B and the yellow line indicates the sequences identified as *Trypanosoma* sp. DID.

**Figure 3:**
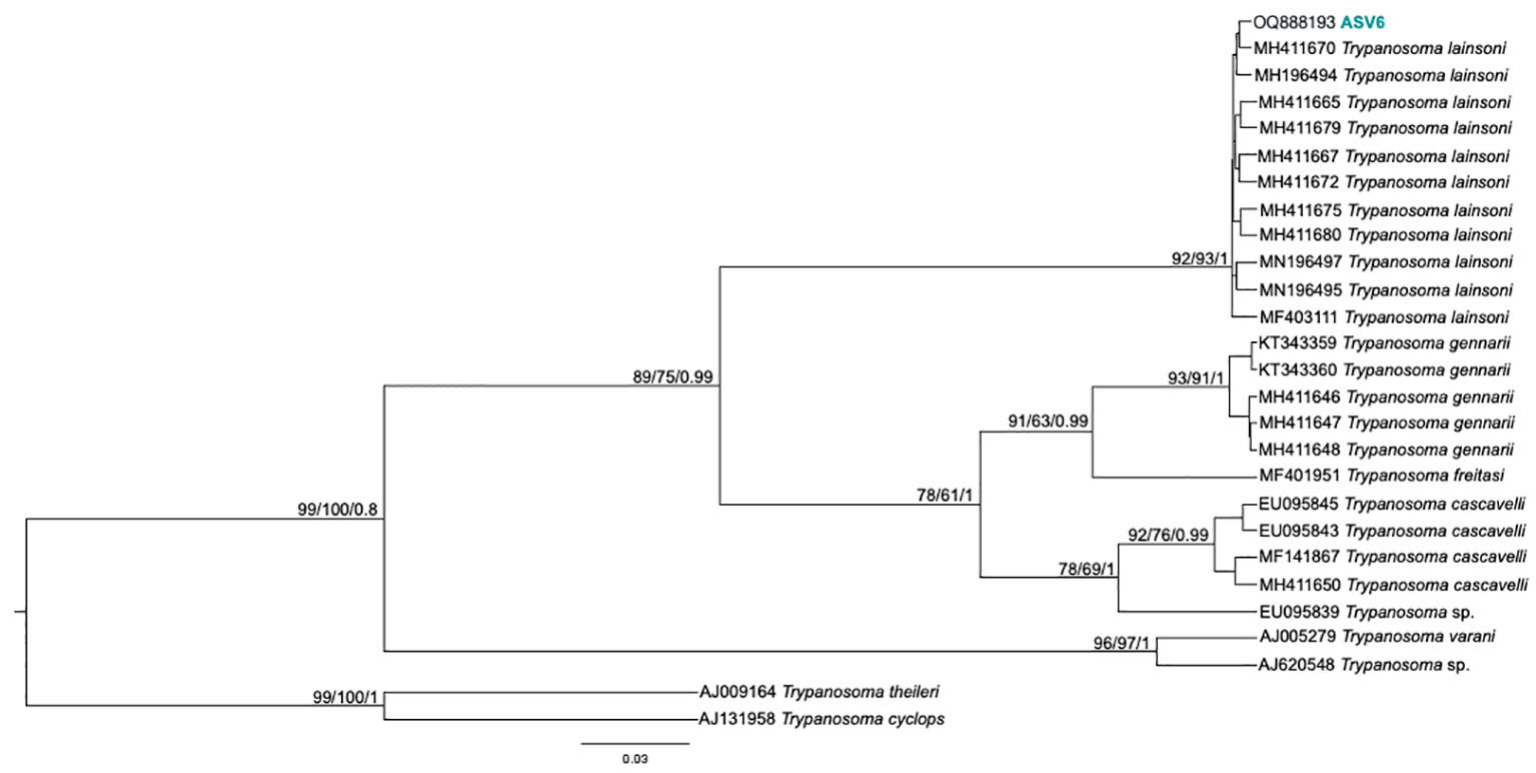
*Trypanosoma lainsoni* phylogenetic tree based on 18S rDNA gene from whole blood DNA from different primate species. Phylogenetic tree was inferred with Tamura Nei model plus gamma distribution among sites (TN+G). The number at nodes corresponds to SH-aLRT, ultrabootstrap (ML) and posterior probability (BI), respectively. The scale bar shows the number of nucleotide substitutions per site. The ASV6 grouped with *T. lainsoni* sequences from different mammal species and biomes.

**Figure 4:**
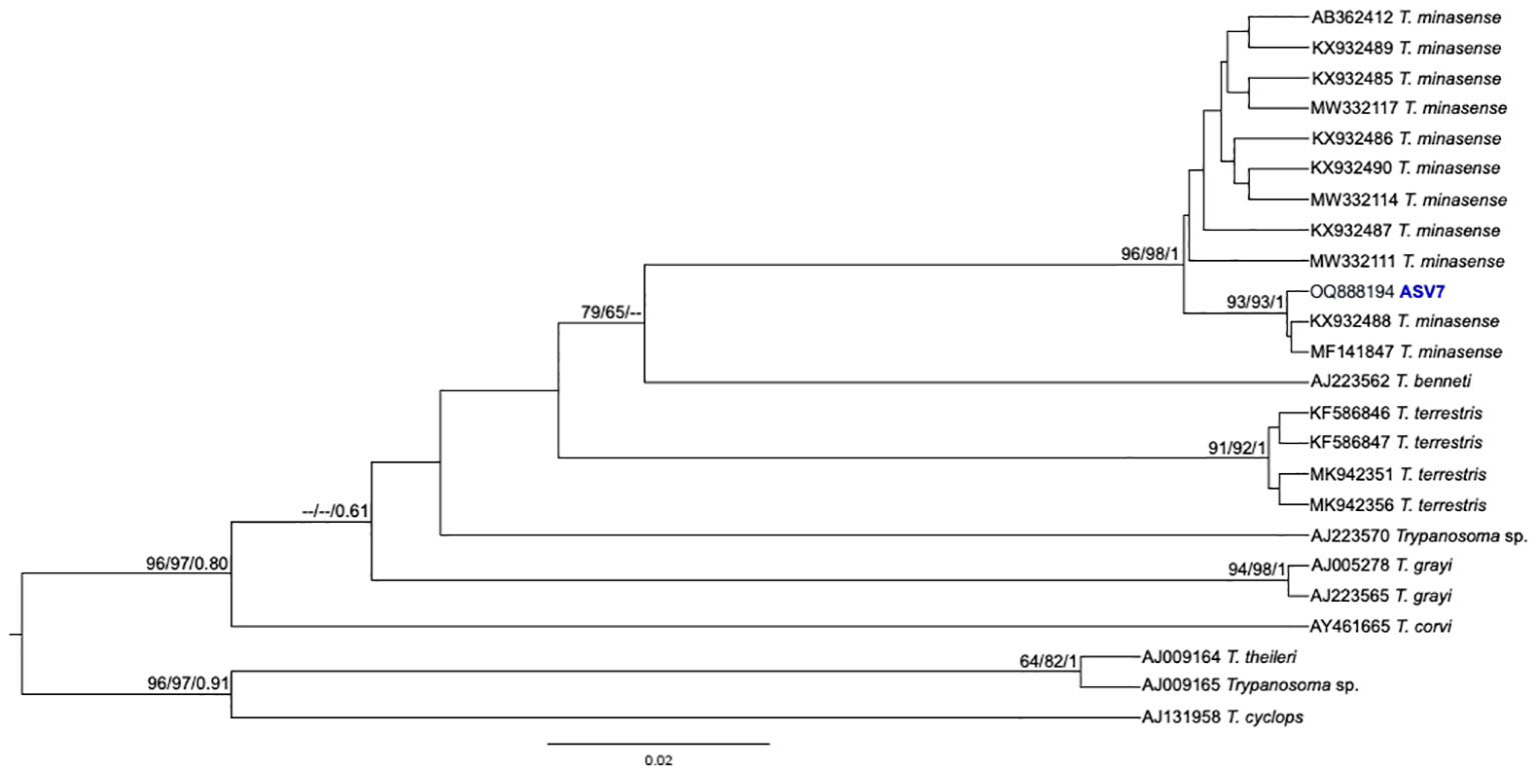
*Trypanosoma minasense* phylogenetic tree based on 18S rDNA gene from whole blood DNA from different primate species. Phylogenetic tree was inferred with the transition model with invariant, equal frequency plus gamma distribution among sites (TIMef3+I+G). The number at nodes corresponds to SH-aLRT, ultrabootstrap (ML) and posterior probability (BI), respectively. The scale bar shows the number of nucleotide substitutions per site. The ASV7 grouped with *T. minasense s*equences.

**Figure 5:**
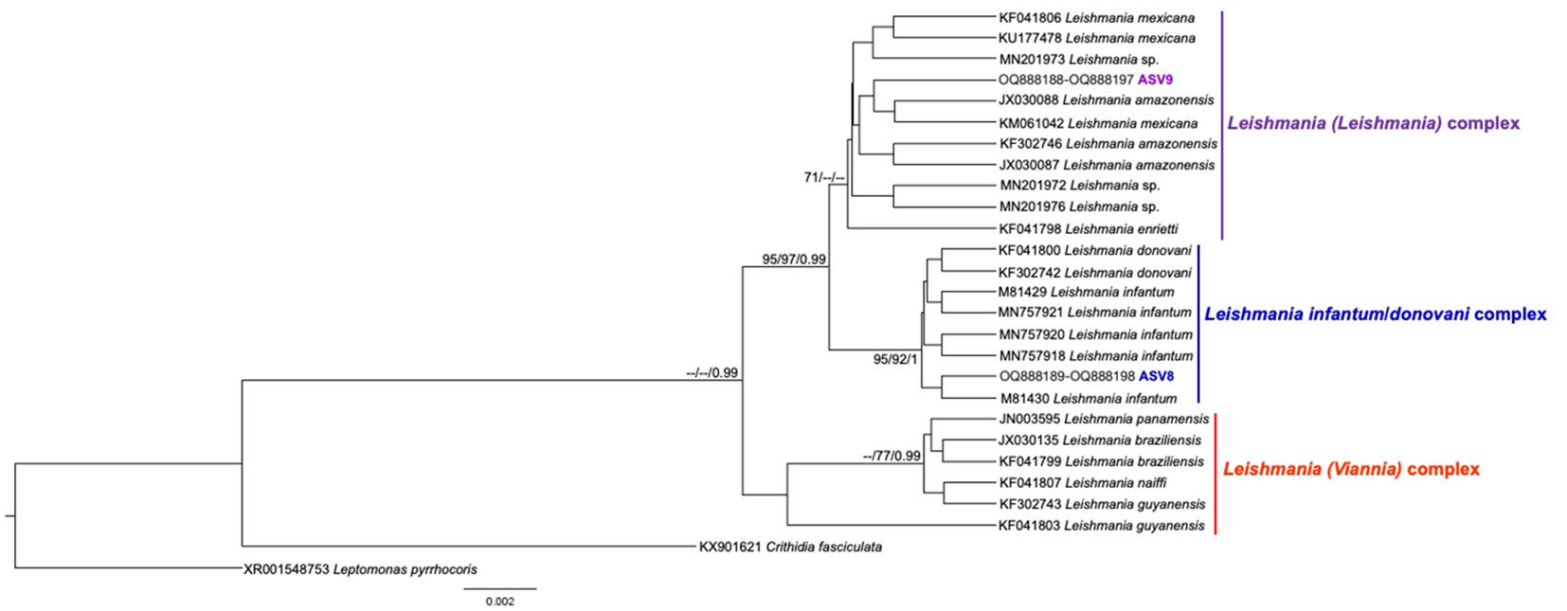
*Leishmania* spp. phylogenetic tree based on 18S rDNA gene from whole blood DNA from different primate species. Phylogenetic tree was inferred with the Tamura Nei equal site frequencies model plus gamma distribution among sites (TNef3 + G). The number at nodes corresponds to SH-aLRT, ultrabootstrap (ML) and posterior probability (BI), respectively. The scale bar shows the number of nucleotide substitutions per site. The purple, blue, and red lines correspond to the *Leishmania* (*Leishmania*), *L.* (*L.*) *infantum/donovani*, and *L.* (*Viannia*) complexes, respectively. The ASV9 grouped with *Leishmania* sequences from the *Leishmania* (*Leishmania*) complex, identified by geographical criteria as *Leishmania* (*L.*) *amazonensis*, and the ASV8 grouped with *Leishmania* sequences from the *Leishmania* (*L.*) *infantum* complex.

**Figure 6:**
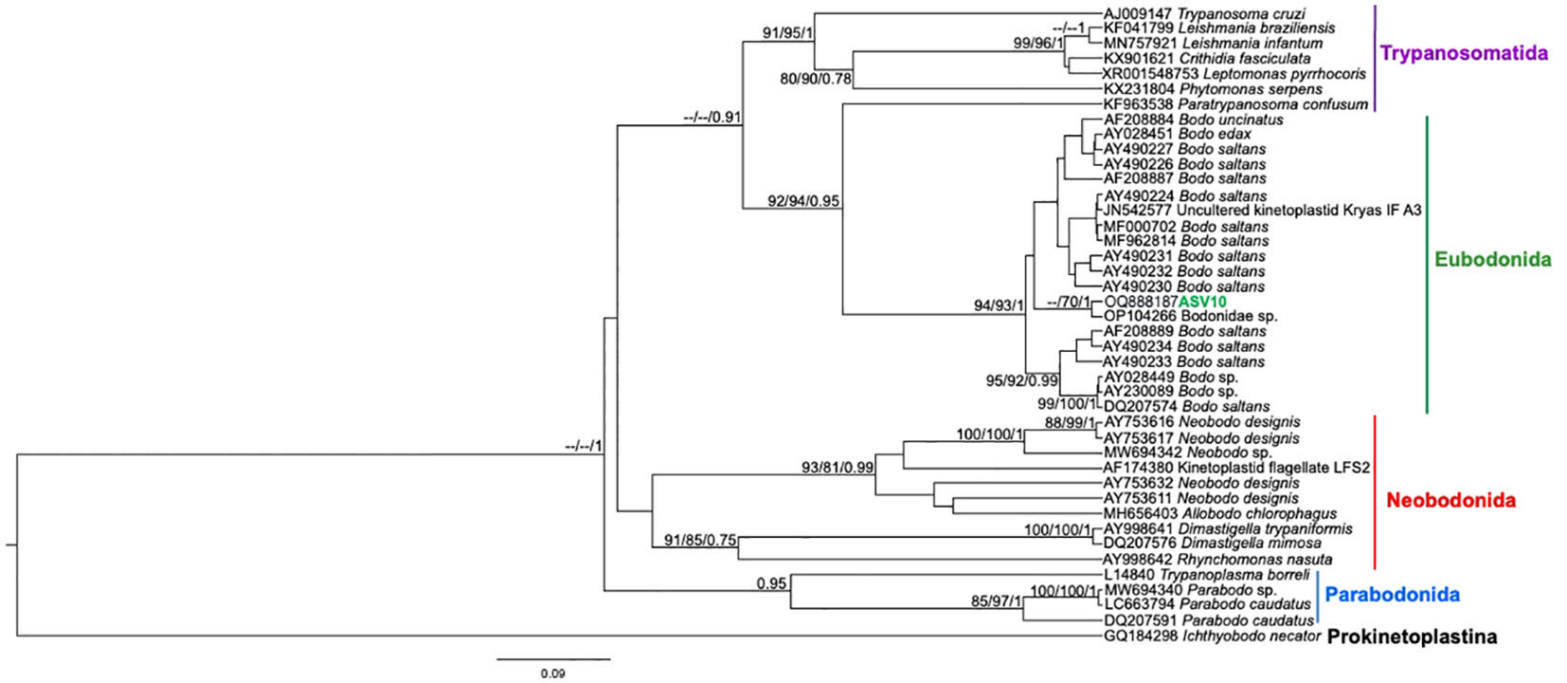
Kinetoplastea class phylogenetic tree based on 18S rDNA gene from whole blood DNA from different primate species. Phylogenetic tree was inferred with the SYM model plus gamma distribution among sites (SYM+G). The number at nodes corresponds to SH-aLRT, ultrabootstrap (ML) and posterior probability (BI), respectively. The line colors correspond to the following trypanosomatid orders: purple – Trypanosomatida; green – Eubodonida; red- Neobonida; blue – Parabodonida. The ASV10 grouped together.

From the nPCR positive samples, we selected the 32 that presented a minimum concentration of 400 ng for NGS analysis: four from ISV, 16 (15 individuals and 1 recapture) from PEMS, and 12 from WRC (**Table 1**). A summary of the NGS sequencing data quality is shown in **Supplementary Table 1**. A total of 3,330,184 raw sequences were obtained. Then, 2,548,089 reads were selected after the preliminary quality filtering. Denoised F/R quality filtering was then performed, which yielded 2,520,437 (denoised F) and 2,519,297 (denoised R) reads. The total number of merged forward-reverse reads was 2,353,170 sequences, and 2,267,671 sequences were selected for analysis after chimera removal. Finally, the relative number of passed reads after all the above steps ranged from 94.46 to 99.68%. In the 436 reads that constituted the database, we observed ten groups of kinetoplastids, according to the phylogenetic analysis: *T. cruzi* Discrete Typing Unit (DTU) TcI (ASV2/GenBank accession number OQ888191), *T. cruzi* DTU TcII/VI (ASV3/GenBank accession number OQ888192 and OQ888200), *T. cruzi* DTU TcIV (ASV1/GenBank accession number OQ888190 and OQ888199), *T. rangeli* lineage B (ASV4/GenBank accession number OQ888195), *Trypanosoma* sp. DID (ASV5/GenBank accession number OQ888196 and OQ888202) (**Figure 2**); *T. lainsoni* (ASV6/GenBank accession number OQ888193) (**Figure 3**); *T. minasense* (AVS7/GenBank accession number OQ888194 and OQ888201) (**Figure 4**); *L. (L.) infantum* (ASV8/GenBank accession number OQ888189 and OQ888198); *L. (L.) amazonensis* (ASV9/GenBank accession number OQ888188 and OQ888197) (**Figure 5**); and *Bodo* sp. (AVS10/GenBank accession number OQ888187) (**Figure 6**).

Co-infections were more predominant (n = 30) than single infections. Indeed, only two females of capuchin monkeys sampled in PEMS displayed single infections by *Trypanosoma* sp. DID (**Table 1**). Concerning capuchin monkeys (n = 28 samples), the following infections were detected in the sampled areas: *Trypanosoma* sp. DID in all examined samples (n = 28), *L. infantum* (n = 22), *T. cruzi* TcII (n = 18) and *L. amazonensis* (n = 4) (**Table 1**). We found 12 individuals infected by *T. cruzi* TcI, nine in the conserved PEMS and three in the anthropized ISV. Concerning *T. cruzi* TcIV, we detected seven capuchin monkeys in PEMS and four in WRC. We also detected *T. minasense* in the PEMS (n = 9) and WRC (n = 4). Furthermore, *T. rangeli* (n = 2) and *Bodo* sp. (n = 1) were detected only in ISV and *T. lainsoni* (n = 1) only in WRC (**Table 1**). The female 304 captured in PEMS in December 2020 (ID number 304A) showed infection by *T. cruzi* TcI, TcII and TcIV, besides *Trypanosoma* sp. DID in the clot, and nine months later (ID number 304B) this animal displayed only *Trypanosoma* sp. DID, positive in whole blood (**Table 1**). Additionally, a male captured in ISV (ID number 489) was positive for *T. rangeli* in axenic culture (GenBank access number ON364108) and in the NGS, also this animal showed coinfection with *L. infantum*, *L. amazonensis* and *Trypanosoma* sp. DID (**Table 1**).

All Howler monkeys (n = 4) displayed coinfections by *T. cruzi* (three by TcIV and one by TcII/TcIV), *T*. *minasense*, and *Trypanosoma* sp. DID. Also, we detected *L. amazonensis* (n = 2) and *L. infantum* (n = 2) in Howler monkey, always in association with other trypanosomatids (**Table 1**). Concerning *T. cruzi* infection, we highlight the *T. cruzi* TcIV that was found in all four Howler monkeys and 11 capuchin monkeys. This genotype was detected separately (n = 7) or together with TcI and TcII (n = 8) (**Table 1**).

## 4 Discussion

We detected DNA sequences of ten kinetoplastid species and *T. cruzi* genotypes in the NHP that inhabit urban forested fragments at Campo Grande, including for the first time *T. lainsoni* and *Trypanosoma* sp. DID, originally associated with small mammal species (Rodrigues et al., 2019; Nantes et al., 2021; Santos et al., 2022), and *Bodo* sp., a putative free-living protozoon. The other detected species *L. (L.) infantum*, *L. (L.) amazonensis*, *T. cruzi* TcI, TcII/TcVI and TcIV, *T. minasense* and *T. rangeli*, these last also isolated, already have been recorded infecting primates in the Latin America (Acosta et al., 2016; Cândido et al., 2021; Guiraldi et al., 2022). The great richness of kinetoplastids found in this study reflects the ability of the capuchin and the Howler monkey to inhabit different ecological niches (soil, understory and canopy) and feed on both insects (potential vectors) and small mammals (Porfirio et al., 2017; Williamson et al., 2021). Besides this huge diversity and detection rates, the active transmission in the studied area is attested by the five recapture events, including four animals that were positive in both sampling in an interval up to nine months, and a NHP that was negative in the first sampling, but positive one year later.

As different trypanosomatid species present differences in their biology, inhabiting different tissues, the use of different templates, such as total blood, blood clot, and bone marrow increased our detection, and no association was observed between a particular trypanosomatid species and the host tissue. We did not find differences according to sex regarding the detection of trypanosomatids. Even though in nature male and female of NHP are reported to forage in different stratum (Williamson et al., 2021), probably the anthropized environments can modify the natural behavior of NHP (McLennan et al., 2017).

The kinetoplastid detections performed by the analysis of small DNA reads (200bp – 400bp) obtained by NGS, especially in the case of *T. cruzi* and *Leishmania* spp. in animals that were seronegative for these parasites, led us wonder whether the detection of these DNA fragments could (or not) represent infection in these animals. Seronegative animals with blood circulating DNA from *T. cruzi* and *Leishmania* spp. represent a challenge that still needs to be clarified. It would be difficult to think that everyone would be at the beginning of infection (immunological window), as reported in natural and experimental infections by trypanosomatids (Herrera et al., 2001; Herrera et al., 2004; Rodrigues et al., 2019). We can interpret the seronegativity as no success in the multiplication of the parasites and consequently failure in the establishment of infection and, consequently, production of IgG. We also considered the possibility of protein degradation in the serum that would lead to non-detection of immunoglobulins in the performed tests. However, some individuals presented positive IFAT or ELISA reactions, but none of them in both tests, which would be necessary to consider these animals seropositive. In addition, as a quality control step, we submitted these sera to a immunocromatographic Chagas disease test (TR Chagas, Bio-Manguinhos^®^, Rio de Janeiro, Brazil), and the positivity in the control band attested the integrity of the sera, since the protein A of the test was able to bind to the preserved fraction of the immunoglobulin (FC fraction) present in the samples (data not shown). One possibility is that, after *T. cruzi* and *Leishmania* spp. parasites penetrate in the NHP host, by vectorial or oral route, they are destroyed by mechanisms distinct of the humoral immunological response, through mechanisms to be elucidated. The herein detection, in this case, would be the result of small DNA sequences (reeds) found in suspension in the circulatory system because NGS is a very sensitive tool capable of detecting nucleic acid sequences at a concentration of 0.0016 ng (less than 1 equivalent parasite) (Nasereddin et al., 2022).

In the case of *Leishmania* sp., although neotropical primates have been suggested as hosts and/or potential reservoirs (Roque and Jansen, 2014; Santos and de Oliveira, 2020), and captive NHP has been reported to infect *Lu. longipalpis* with *L. infantum* (Rodrigues de Oliveira et al., 2019), the NHP resistance to *Leishmania* infection has already been reported by experimental and natural infections. In fact, resistance to infection can be described as limiting the infection burden itself, a defense strategy developed throughout the evolutionary process (Råberg et al., 2009). Carneiro et al. (2012) examined the susceptibility *in vitro* of some neotropical NHP species to *L. infantum*, suggesting that they developed an efficient mechanism capable of controlling the macrophage intracellular growth of *L. infantum*. Based in their results, these authors do not encourage the use of neotropical NHP as an animal model for studying visceral leishmaniasis. The control of infection by *L. amazonensis*, *L*. *braziliensis* and *L*. *lainsoni* in *S. apella* experimentally infected was associated to the ability of different species of *Leishmania* to trigger both innate and acquired immune responses that allow elimination of the parasite (Silveira et al., 2004; Laurenti et al., 2014).

Considering their possible role as dead-end hosts for *Leishmania* sp., *S. cay* and *A. caraya* that inhabit urban forest fragments in CG may have an important epidemiological role because, as the area is considered of intense transmission of visceral leishmaniasis for humans and dogs (Botelho and Natal, 2009; Brazuna et al., 2012; Cunha et al., 2014), every time that an infected vector feeds on *S. cay* and *A. caraya*, the transmission cycle of *L. infantum* will be largely reduced or interrupted. Contemplating the hypothesis that *S. cay* and *A. caraya* can be considered as dead-end hosts for *Leishmania* species in CG, they may have an important role in the local reservoir system by promoting an ecological phenomenon known as the dilution effect. It is worth mentioning, however, that future studies involving xenodiagnosis and characterization of skin parasitism is necessary to confirm the role of these primates as dead-end *Leishmania* hosts in CG (Bourdeau et al., 2020).

The non-colonization by *T. cruzi* and *Leishmania* spp. may also be related to the anointing behavior in NHP, characterized by crushing and rubbing parts of plants, citrus fruits, mud and insects in their body (Baker, 1996; Verderane et al., 2007). The genus *Piper*, a pioneer and invasive plant found throughout in the forest fragments of CG (Lepš et al., 2002; Kukla et al., 2022), has been reported *in vitro* to have strong activities against *Leishmania* (Garcia et al., 2013; Ceole et al., 2017) and *Trypanosoma* (Regasini et al., 2009; Villamizar et al., 2017). Since it has been recorded that NHP use plant parts for self-medication in a preventive or therapeutic way (De la Fuente et al., 2022), and it is reported that NHP eat and rub *Piper* (Gonçalves et al., 2022), this association would be related to the non-success of trypanosomatid colonization. Indeed, search for *T. cruzi* in primates that inhabit urban forest fragments in Northeast Brazil (by PCR and hemoculture) did not find positive results (Trüeb et al., 2018). However, this hypothesis cannot be considered a general rule for a given population since *T. rangeli* was isolated from a single *S. cay* in this study. We highlight that elevated parasitemia by *T. cruzi*, expressed by positive hemoculture, is observed in NHP only those inhabiting megadiverse environments such as the Amazon and Atlantic Forest, and not in urban environments (Jansen et al., 2020).

The observed results in the NHP from CG indicate that TcI, TcII/TcVI and TcIV genotypes of *T. cruzi* circulate in the studied area. Sympatric lineages of *T. cruzi* have occurred in transmission cycles independent of their hosts (Zingales and Bartholomeu, 2022). Different genotypes such as TcI, TcII, TcIII, TcV and TcVI were found infecting NHP of the genus *Alouatta* and *Ateles* in southern Mexico (Rovirosa-Hernández et al., 2021) and TcI and TcIV genotypes have been reported infecting captive primates at one of the National Primate Center facilities in Texas (USA) (Hodo et al., 2018).

Here, we increase knowledge about the geographical distribution of *T. cruzi* TcIV, not previously reported in CG. In the Brazilian Amazon, several NHP species were reported infected with TcI and TcIV, with the most common TcI genotype associated with *Rhodnius* species (Marcili et al., 2009), already recorded in CG (Santos FM personal communication). Also, Abad-Franch and Gurgel-Gonçalves (2021) mentioned that *Triatoma sordida* could act as a vector of *T. cruzi* to howler monkeys in the humid Chaco and Paraná flooded savanna. Moreover, we found the DTU TcIV in single or mixed infection with TcI and/or TcII/TcVI in *S. cay* and *A. caraya*, as previously reported in humans, reduviid species and wildlife such as bats, rodents and marsupials throughout several Brazilian biomes (Rodrigues et al., 2017; dos Santos et al., 2018; Silva et al., 2022). The DTU TcIV is associated with the sylvatic cycle, commonly found in arboreal stratum (Barros et al., 2017), and terrestrial microhabitats (Abad-Franch and Gurgel-Gonçalves, 2021). As Cebidae species can also go to the ground in search of fruits and insects (Porfirio et al., 2017), they may also act as a bridge among transmission cycles in different forest strata (Jansen et al., 2017).

We found NHP infected by *T. cruzi* TcI and TcII genotypes in all sampled areas. Specifically in CG, TcI and TcII were previously reported in bats and opossums *D. albiventris*, respectively, that inhabit green areas (Nantes et al., 2021; Torres et al., 2024). Also, de Macedo GC (manuscript in preparation) found TcI and TcII infecting coatis *N. nasua* that inhabiting green areas of CG. These genotypes are commonly found in Latin America in rodents, marsupials, bats, carnivores and primates, without any association with biological, environmental variable (biomes) or host species (Jansen et al., 2020; Gómez-Sánchez et al., 2022). Moreover, the golden-lion tamarins (*Leontopithecus rosalia* and *Leontopithecus chrysomelas*) are suggested as reservoirs of TcII in Atlantic Rain Forest (Lisboa et al., 2015).

We detected *T. minasense* in 37.5% of the individuals sampled at PEMS and WRC, both forested areas. This could suggest that forest cover could favor the interaction with possible infected vectors. *T. minasense* was first described infecting black-tufted marmosets (*C. penicillata*) in Brazil (Rodhain, 1941; Deane et al., 1974), and it has been documented infecting marmosets, capuchins, squirrel monkeys, spider monkeys, howler monkeys, and wooly monkeys from Central America to South America (Deane et al., 1974; Sousa and Dawson, 1976; Ziccardi et al., 2000); however, their developmental stages and its vector remain unknown. It has been reported that NHP in captivity has been already found parasitizing by *T. minasense* (Malta et al., 2010; Guiraldi et al., 2022), probably due to the long duration of infection, they may arrive in captive already infected from the wild (Rodhain, 1941; Deane et al., 1974).

In the ISV, an unprotected area under pressure from anthropogenic activities, we found a *S. cay* that displayed high parasitemia for *T. rangeli* (indicative of an active transmission). This animal also displayed co-infection with *L. infantum*, *L. amazonensis* and *Trypanosoma* sp. DID. In this area, we also detected another animal infected with *T. rangeli*, together with TcI and TcII/TcVI, *L. infantum* and *Trypanosoma* sp. DID. The *T. cruzi* and *T. rangeli* co-infection is not uncommon among free-living mammal species and triatomines (Maia da Silva et al., 2008; Jansen et al., 2018; Santos et al., 2019).

*Trypanosoma* sp. DID detected in all sampled NHP shows that this Molecular Operational Taxonomic Unit (MOTU) is quite dispersed in NHP, regardless of its transmission strategy. Rodrigues et al. (2019) showed that *Trypanosoma* sp. DID is a MOTU positioned in a clade that includes trypanosomes detected in chiropteran hosts. However, this MOTU has been already detected in opossums as *Didelphis* spp. at Atlantic rainforest (Rodrigues et al., 2019; Dario et al., 2022b) and Cerrado biomes (Nantes et al., 2021; Dario et al., 2022b), in didelphid *Thylamys macrurus*, small rodents *Oecomys mamorae* and *Clyomys laticeps* (Santos et al., 2022), as well as in the rattlesnake *C. durissus* in CG, state of Mato Grosso do Sul (Nantes et al., 2024). The fact of finding *Trypanosoma* sp. DID in NHP increases knowledge about the diversity of host species that this uncultured trypanosomatid can parasitize.

The detection of *T. lainsoni* in a single *S. cay* at WRC simply reflects a snapshot and provides no information about the parasitism status but indicates that NHP is exposed to the transmission cycle of this flagellate. Indeed, Nantes et al. (2021) reported *T. laisoni* in opossum *D. albiventris* in the “Parque Estadual do Prosa”, where the WRC is located. Nevertheless, here we increase the knowledge about the host species than previously reported since it has been reported in *Mesomys hispidus* (Echimyidae), the opossum *G. agilis* and *D. albiventris*, and the cricetids *O. mamorae* and *Rhipidomys macrurus* from the Brazilian Amazon rainforest, Cerrado and Pantanal biomes (Naiff and Barrett, 2013; Rodrigues et al., 2019; Nantes et al., 2021; Santos et al., 2022; de Oliveira et al., 2023). Many ecological aspects of *T. lainsoni* are still unknown, as its vector, and there was a previous unsuccessful attempt at experimental infection of *T. lainsoni* in laboratory rodents, *D. marsupialis*, and triatomine vectors (Naiff and Barrett, 2013; de Oliveira et al., 2023).

Because *Bodo* sp., a free-living bacterivorous kinetoplastid closest relative of the trypanosomatid parasites, are found worldwide in freshwater habitats (Mitchell et al., 1988; Jackson et al., 2008), we can consider the possibility that their ASVs can be found in the bloodstream after ingestion of food or water contaminated. It has been reported that meal-derived DNA fragments can resist the digestive process and, through an unknown mechanism, cross the intestinal barrier into the circulatory system (Spisák et al., 2013). Indeed, there are animal studies supporting the idea that small sequences of nucleic acids can pass directly into the bloodstream and even enter various tissues (Rizzi et al., 2012). Despite that, the possibility of an active infection, previously suggested for bats (Alves et al., 2023), cannot be ruled out.

Our results show that the forest fragments in CG maintain diverse kinetoplastid transmission cycles, as we found DNA sequences of *L. infantum*, *L. amazonensis*, *T. cruzi*, *T. minasense*, *T. rangeli, T. lainsoni, Trypanosoma* sp. DID, and *Bodo* sp. in the sampled NHPs. In relation to *T. lainsoni* and *Trypanosoma* sp. DID, the NHP indicates that the transmission cycle also occurs in the arboreal stratum. Furthermore, NHPs living in forest fragments at CG have been shown to be, at least, exposed to the reservoir system of highly important zoonotic parasites for Public Health, such as *L. infantum*, *L. amazonensis* and *T. cruzi*.

## Data Availability

The datasets presented in this study can be found in online repositories. The names of the repository/repositories and accession number(s) can be found in the article/**Supplementary Material**.

## References

[B1] Abad-Franch F. Gurgel-Gonçalves R. (2021). “The Ecology and Natural History of Wild Triatominae in the America,” in Triatominae - The Biology of Chagas Disease Vectors. Entomology in Focu. Eds. Guarneri A. Lorenzo M. (Springer, Cham, Cham), 387–445.

[B2] Acosta N. Miret J. López E. Schinini A. (2016). First report of *Sapajus cay* naturally infected by *Trypanosoma cruzi* in San Pedro Department, Paraguay. Rev. Bras. Parasitol. Vet. 25, 327–332. doi: 10.1590/S1984-29612016052 27579529

[B3] Alfaro J. W. L. Matthews L. Boyette A. H. Macfarlan S. J. Phillips K. A. Falótico T.. (2012). Anointing variation across wild capuchin populations: a review of material preferences, bout frequency and anointing sociality in *Cebus* and Sapajus. Am. J. Primatol 74, 299–314. doi: 10.1002/ajp.20971 21769906

[B4] Alves F. Lisboa C. Dario M. Novaes R. Tiepolo L. Moratelli R.. (2023). Old methods, new insights: reviewing concepts on the ecology of trypanosomatids and *Bodo* sp. by improving conventional diagnostic tools. Pathogens 12, 71. doi: 10.3390/pathogens1201007 36678419 PMC9864408

[B5] Back J. Suzin A. Aguiar L. (2019). Activity budget and social behavior of urban capuchin monkeys, *Sapajus* sp. (Primates: Cebidae). Zoologia 36, 1–10. doi: 10.3897/zoologia.36.e30845

[B6] Baker M. (1996). Fur rubbing: Use of medicinal plants by capuchin monkeys (*Cebus capucinus*). Am. J. Primatol. 38, 263–270. doi: 10.1002/(SICI)1098-2345(1996)38:3<263::AID-AJP5>3.0.CO;2-X

[B7] Barbosa A. D. Gofton A. W. Paparini A. Codello A. Greay T. Gillett A.. (2017). Increased genetic diversity and prevalence of co-infection with *Trypanosoma* spp. in koalas (*Phascolarctos cinereus*) and their ticks identified using next-generation sequencing (NGS). PloS One 12, e0181279. doi: 10.1371/journal.pone.0181279 28704541 PMC5509321

[B8] Barros J. H. S. Xavier S. C. C. Bilac D. Lima V. S. Dario M. A. Jansen A. M. (2017). Identification of novel mammalian hosts and Brazilian biome geographic distribution of *Trypanosoma cruzi* TcIII and TcIV. Acta Trop. 172, 173–179. doi: 10.1016/j.actatropica.2017.05.003 28499908

[B9] Botelho A. C. A. Natal D. (2009). Primeira descrição epidemiológica da leishmaniose visceral em Campo Grande, Estado de Mato Grosso do Sul. Rev. Soc. Bras. Med. Trop. 42, 503–508. doi: 10.1590/S0037-86822009000500006 19967231

[B10] Bouckaert R. Vaughan T. G. Barido-Sottani J. Duchêne S. Fourment M. Gavryushkina A.. (2019). BEAST 2.5: An advanced software platform for Bayesian evolutionary analysis. PloS Comput. Biol. 15, e1006650. doi: 10.1371/journal.pcbi.1006650 30958812 PMC6472827

[B11] Bourdeau P. Rowton E. Petersen C. (2020). Impact of different *Leishmania* reservoirs on sand fly transmission: Perspectives from xenodiagnosis and other one health observations. Vet. Parasitol. 287, 109237. doi: 10.1016/j.vetpar.2020.109237 33160145 PMC8035349

[B12] Brazuna J. C. M. Silva E. Brazuna J. M. Domingos I. H. Chaves N. Honer M. R.. (2012). Profile and geographic distribution of reported cases of visceral leishmaniasis in Campo Grande, State of Mato Grosso do Sul, Brazil, from 2002 to 2009. Rev. Soc. Bras. Med. Trop. 45, 601–606. doi: 10.1590/S0037-86822012000500012 23152344

[B13] Callahan B. J. McMurdie P. J. Rosen M. J. Han A. W. Johnson A. J. A. Holmes S. P. (2016). DADA2: High-resolution sample inference from Illumina amplicon data. Nat. Methods 13, 581–583. doi: 10.1038/nmeth.3869 27214047 PMC4927377

[B14] Camargo M. E. (1966). Fluorescent antibody test for the serodiagnosis of American trypanosomiasis. Technical modification employing preserved culture forms of *Trypanosoma cruzi* in a slide test. Rev. Inst Med. Trop. 8, 227–234. 4967348

[B15] Cândido S. L. Pavelegini L. A. D. Pacheco T.d. A. Pacheco R.de C. Silva V. L. de B. Morgado T. O.. (2021). Molecular detection of trypanosomatids in neotropical primates in the state of Mato Grosso, Midwest, Brazil. Rev. Bras. Parasitol. Vet. 30, e00132. doi: 10.1590/s1984-29612021041 34076047

[B16] Carneiro L. A. Laurenti M. D. Campos M. B. Gomes C. M. de C. Corbett C. E. P. Silveira F. T. (2012). Susceptibility of peritoneal macrophage from different species of neotropical primates to Ex vivo Leishmania (L.) infantum chagasi-infection. Rev. Inst Med. Trop. Sao Paulo 54, 95–102. doi: 10.1590/S0036-46652012000200007 22499423

[B17] Castro L. S. Dorval M. E. C. Matheus L. M. D. Bednaski A. V. Facco G. G. Silveira M.. (2020). *Leishmania* presence in bats in areas endemic for leishmaniasis in central-west Brazil. Int. J. Parasitol. Parasites Wildl. 11, 261–267. doi: 10.1016/j.ijppaw.2020.02.008 32195111 PMC7078454

[B18] Ceole L. F. Cardoso M das G. Soares M. J. (2017). Nerolidol, the main constituent of *Piper aduncum* essential oil, has anti- *Leishmania Braziliensis* activity. Parasitology 144, 1179–1190. doi: 10.1017/S0031182017000452 28482935

[B19] Chaves Ó. M. Fortes V. B. Hass G. P. Azevedo R. B. Stoner K. E. Bicca-Marques J. C. (2021). Flower consumption, ambient temperature and rainfall modulate drinking behavior in a folivorous-frugivorous arboreal mammal. PloS One 16, e0236974. doi: 10.1371/journal.pone.0236974 33606693 PMC7894884

[B20] Cibot M. Guillot J. Lafosse S. Bon C. Seguya A. Krief S. (2015). Nodular worm infections in wild non-human primates and humans living in the Sebitoli area (Kibale National Park, Uganda): Do High Spatial Proximity Favor Zoonotic Transmission? PloS Negl. Trop. Dis. 9, e0004133. doi: 10.1371/journal.pntd.0004133 26451592 PMC4599739

[B21] Cunha R. C. Andreotti R. Cominetti M. C. Silva E. A. (2014). Detection of *Leishmania infantum* in *Lutzomyia longipalpis* captured in Campo Grande, MS. Rev. Bras. Parasitol. Vet. 23, 269–273. doi: 10.1590/S1984-29612014049 25054512

[B22] Dario M. A. Furtado C. Lisboa C. V. de Oliveira F. Santos F. M. D’Andrea P. S.. (2022a). Trypanosomatid richness among rats, opossums, and dogs in the Caatinga Biome, Northeast Brazil, a Former Endemic Area of Chagas Disease. Front. Cell Infect. Microbiol. 12. doi: 10.3389/fcimb.2022.851903 PMC9251133 35795183

[B23] Dario M. A. Lisboa C. V. Xavier S. C. d. C. D’Andrea P. S. Roque A. L. R. Jansen A. M. (2022b). Trypanosoma species in small nonflying mammals in an area with a single previous chagas disease case. Front. Cell Infect. Microbiol. 12. doi: 10.3389/fcimb.2022.812708 PMC8873152 35223545

[B24] Darriba D. Taboada G. L. Doallo R. Posada D. (2012). jModelTest 2: more models, new heuristics and parallel computing. Nat. Methods 9, 772–772. doi: 10.1038/nmeth.210 PMC4594756 22847109

[B25] da Silva A. R. Herrera H. M. de Oliveira C. E. Torres J. M. Ferreira A. M. R. Leite J da S.. (2024). The relationships among *Leishmania infantum* and phyllostomid bats assessed by histopathological and molecular assays. Int. J. Parasitol. Parasites Wildl. 23, 100904. doi: 10.1016/j.ijppaw.2023.100904 38261956 PMC10797179

[B26] de-Almeida-Rocha J. M. Peres C. A. Oliveira L. C. (2017). Primate responses to anthropogenic habitat disturbance: A pantropical meta-analysis. Biol. Conserv. 215, 30–38. doi: 10.1016/j.biocon.2017.08.018

[B27] Deane L. M. da Silva J. E. Loures Filho L. (1974). Nycthemeral variation in the parasitaemia of *Trypanosoma minasense* in naturally infected marmosets of the genus *Callithrix* (Primates, Callithricidae). Rev. Inst Med. Trop. Sao Paulo. 16, 1–6. 4210841

[B28] De la Fuente M. F. Souto A. Albuquerque U. P. Schiel N. (2022). Self-medication in nonhuman primates: A systematic evaluation of the possible function of the use of medicinal plants. Am. J. Primatol. 84, e23438. doi: 10.1002/ajp.23438 36193566

[B29] de Macedo G. C. Barreto W. T. G. de Oliveira C. E. Santos F. M. Porfírio Ge de O. Xavier S. C. d. C.. (2023). *Leishmania infantum* infecting the carnivore *Nasua nasua* from urban forest fragments in an endemic area of visceral leishmaniasis in Brazilian Midwest. Front. Cell Infect. Microbiol. 12. doi: 10.3389/fcimb.2022.1050339 PMC9880478 36710973

[B30] de Oliveira M. M. Ferrando C. P. R. Gómez-Hernández C. de Oliveira K. R. Araújo I. A. C. Ribeiro P. V. A.. (2023). Prevalence of *Trypanosoma lainsoni* and its effects of parasitism on the health of non-volant small mammals from the Brazilian Cerrado. Parasitol. Res. 122, 1509–1518. doi: 10.1007/s00436-023-07851-1 37129625

[B31] de Rezende M. B. Herrera H. M. Carvalho C. M. E. Carvalho Anjos E. A. Ramos C. A. N. de Araújo F. R.. (2017). Detection of *Leishmania* spp. in bats from an area of Brazil endemic for visceral leishmaniasis. Transbound Emerg. Dis. 64, e36–e42. doi: 10.1111/tbed.12597 28233434

[B32] Dian N. D. Rahim M. A. F. A. Chan S. Idris Z. M. (2022). Non-human primate malaria infections: A review on the epidemiology in Malaysia. Int. J. Environ. Res. Public Health 19, 7888. doi: 10.3390/ijerph19137888 35805545 PMC9265734

[B33] Do Nascimento F. F. Bonvicino C. R. Seuánez H. N. (2007). Population genetic studies of *Alouatta caraya* (Alouattinae, Primates): inferences on geographic distribution and ecology. Am. J. Primatol. 69, 1093–1104. doi: 10.1002/ajp.20423 17330870

[B34] dos Santos F. C. B. Lisboa C. V. Xavier S. C. C. Dario M. A. Verde R de S. Calouro A. M.. (2018). *Trypanosoma* sp. diversity in Amazonian bats (Chiroptera; Mammalia) from Acre State, Brazil. Parasitology 145, 828–837. doi: 10.1017/S0031182017001834 29144219

[B35] Estrada A. Garber P. A. Rylands A. B. Roos C. Fernandez-Duque E. Di Fiore A.. (2017). Impending extinction crisis of the world’s primates: Why primates matter. Sci. Adv. 3, e1600946. doi: 10.1126/sciadv.1600946 28116351 PMC5242557

[B36] Falótico T. (2023). Vertebrate predation and tool-aided capture of prey by savannah wild capuchin monkeys (*Sapajus libidinosus*). Int. J. Primatol. 44, 9–20. doi: 10.1007/s10764-022-00320-z

[B37] Garcia F. P. Lazarin-Bidóia D. Ueda-Nakamura T. Silva S.de O. Nakamura C. V. (2013). Eupomatenoid-5 isolated from leaves of *Piper regnellii* induces apoptosis in Leishmania amazonensis. Evid Based Complement Alternat Med. 2013, 1–11. doi: 10.1155/2013/940531 PMC3618946 23573160

[B38] Gómez-Sánchez E. F. Ochoa-Díaz-López H. Espinoza-Medinilla E. E. Velázquez-Ramírez D. D. Santos-Hernandez N. G. Ruiz-Castillejos C.. (2022). Mini-exon gene reveals circulation of TcI *Trypanosoma cruzi* (Chagas, 1909) (Kinetoplastida, Trypanosomatidae) in bats and small mammals in an ecological reserve in southeastern Mexico. Zookeys 1084, 139–150. doi: 10.3897/zookeys.1084.78664 35177949 PMC8816842

[B39] Gonçalves B.de A. Lima L. C. P. Aguiar L. M. (2022). Diet diversity and seasonality of robust capuchins (*Sapajus* sp.) in a tiny urban forest. Am. J. Primatol. 84, e23396. doi: 10.1002/ajp.23396 35661391

[B40] Guiraldi L. M. dos Santos W. J. Manzini S. Taha N.el H. A. Aires I. N. Ribeiro E.. (2022). Identification of *Leishmania infantum* and *Leishmania Braziliensis* in captive primates from a zoo in Brazil. Am. J. Primatol. 84, e23376. doi: 10.1002/ajp.23376 35384010

[B41] Herrera H. M. Aquino L. P. C. T. Menezes R. F. Marques L. C. Moraes M. A. V. Werther K.. (2001). *Trypanosoma evansi* experimental infection in the South American coati (*Nasua nasua*): clinical, parasitological and humoral immune response. Vet. Parasitol. 102, 209–216. doi: 10.1016/S0304-4017(01)00532-5 11777600

[B42] Herrera H. M. Dávila A. M. R. Norek A. Abreu U. G. Souza S. S. D’Andrea P. S.. (2004). Enzootiology of *trypanosoma evansi* in pantanal, Brazil. Vet. Parasitol. 125, 263–275. doi: 10.1016/j.vetpar.2004.07.013 15482883

[B43] Hoang D. T. Chernomor O. von Haeseler A. Minh B. Q. Vinh L. S. (2018). UFBoot2: improving the ultrafast bootstrap approximation. Mol. Biol. Evol. 35, 518–522. doi: 10.1093/molbev/msx281 29077904 PMC5850222

[B44] Hoare C. A. (1972). The trypanosomes of mammals. A zoological monograph, Vol. 1. (Oxford: Blackwell Scientific Publication).

[B45] Hodo C. L. Wilkerson G. K. Birkner E. C. Gray S. B. Hamer S. A. (2018). *Trypanosoma cruzi* transmission among captive nonhuman primates, wildlife, and vectors. Ecohealth 15, 426–436. doi: 10.1007/s10393-018-1318-5 29497880 PMC6132415

[B46] Humberg R. M. P. Oshiro E. T. Cruz M. d. S. P. e. Ribolla P. E. M. Alonso D. P. Ferreira A. M. T.. (2012). *Leishmania chagasi* in opossums (*Didelphis albiventris*) in an urban area endemic for Visceral Leishmaniasis, Campo Grande, Mato Grosso do Sul, Brazil. Am. J. Trop. Med. Hyg. 87, 470–472. doi: 10.4269/ajtmh.2012.11-0534 22802435 PMC3435349

[B47] Jackson A. P. Quail M. A. Berriman M. (2008). Insights into the genome sequence of a free-living Kinetoplastid: *Bodo saltans* (Kinetoplastida: Euglenozoa). BMC Genomics 9, 594. doi: 10.1186/1471-2164-9-59 19068121 PMC2621209

[B48] Jansen A. M. Xavier S. C. C. Roque A. L. R. (2017). Ecological aspects of Trypanosoma cruzi. Am. Trypanosomiasis Chagas Dis., 243–264. doi: 10.1016/B978-0-12-801029-7.00011-3

[B49] Jansen A. M. Xavier S. C. d. C. Roque A. L. R. (2018). *Trypanosoma cruzi* transmission in the wild and its most important reservoir hosts in Brazil. Parasit Vectors. 11, 502. doi: 10.1186/s13071-018-3067-2 30189896 PMC6127949

[B50] Jansen A. M. Xavier S. C. d. C. Roque A. L. R. (2020). Landmarks of the knowledge and *Trypanosoma cruzi* biology in the wild environment. Front. Cell Infect. Microbiol. 10. doi: 10.3389/fcimb.2020.0001 PMC7016096 32117794

[B51] Júnior O. F. Porfirio G. E. d. O. Santos F. M. Gimenes Nantes W. A. Oliveira de Assis W. Braziliano de Andrade G.. (2020). Behavioral activities and diet of capuchin monkey, *Sapajus cay* (Illiger, 1815), in a forest remnant of the Brazilian Cerrado. Stud. Neotrop. Fauna Environ. 55, 149–154. doi: 10.1080/01650521.2019.1708228

[B52] Kane J. Smith R. L. (2020). *Bertiella* sp. (Meyner, 1895) infection of *Alouatta caraya* (Humboldt, 1812) in urban and natural environments in Ñeembucú, southwest Paraguay. Am. J. Primatol. 82, e23166. doi: 10.1002/ajp.23166 32596875

[B53] Katoh K. Standley D. M. (2013). MAFFT multiple sequence alignment software version 7: improvements in performance and usability. Mol. Biol. Evol. 30, 772–780. doi: 10.1093/molbev/mst01 23329690 PMC3603318

[B54] Kerr C. L. Bhattacharyya T. Xavier S. C. C. Barros J. H. Lima V. S. Jansen A. M.. (2016). Lineage-specific serology confirms Brazilian Atlantic Forest lion tamarins, *Leontopithecus chrysomelas* and *Leontopithecus rosalia*, as reservoir hosts of *Trypanosoma cruzi* II (TcII). Parasit Vectors. 9, 584. doi: 10.1186/s13071-016-1873-y 27846858 PMC5111205

[B55] Kukla J. Heděnec P. Baldrián P. Cajthaml T. Novotný V. Moradi J.. (2022). The invasive tree *Piper aduncum* alters soil microbiota and nutrient content in fallow land following small scale slash-and-burn farming in tropical lowland forest in Papua New Guinea. Appl. Soil Ecol. 176, 104487. doi: 10.1016/j.apsoil.2022.10448

[B56] Kumar S. Stecher G. Tamura K. (2016). MEGA7: molecular evolutionary genetics analysis version 7.0 for bigger datasets. Mol. Biol. Evol. 33, 1870–1874. doi: 10.1093/molbev/msw054 27004904 PMC8210823

[B57] Laurenti M. D. Passero L. F. D. Tomokane T. Y. Francesquini F.de C. Rocha M. C. Gomes C. M. de C.. (2014). Dynamic of the cellular immune response at the dermal site of Leishmania (L.) amazonensis and *Leishmania (V.) Braziliensis* infection in *Sapajus apella* Primate. BioMed. Res. Int. 2014, 1–8. doi: 10.1155/2014/134236 PMC4163356 25309902

[B58] Lepš J. Novotný V. Čížek L. Molem K. Isua B. William B.. (2002). Successful invasion of the neotropical species *Piper aduncum* in rain forests in Papua New Guinea. Appl. Veg Sci. 5, 255–262. doi: 10.1111/j.1654-109X.2002.tb00555.x

[B59] Lisboa C. V. Mangia R. H. Rubião E. de Lima N. R. C. das Chagas Xavier S. C. Picinatti A.. (2004). *Trypanosoma cruzi* transmission in a captive primate unit, Rio de Janeiro, Brazil. Acta Trop. 90, 97–106. doi: 10.1016/j.actatropica.2003.11.005 14739028

[B60] Lisboa C. V. Monteiro R. V. Martins A. F. Xavier S. C. d. C. Lima V.d. S. Jansen A. M. (2015). Infection with *Trypanosoma cruzi* TcII and TcI in free-ranging population of lion tamarins (*Leontopithecus* spp): an 11-year follow-up. Mem. Inst Oswaldo Cruz. 110, 394–402. doi: 10.1590/0074-02760140400 25946156 PMC4489477

[B61] Lopes K. F. C. Delai R. M. Zaniolo M. M. dos Santos I. C. Pachaly E. M. V. Pachaly J. R.. (2022). Urban capuchin monkeys *Sapajus nigritus* (Goldfuss, 1809) (Primates, Cebidae) as environmental bioindicators of leishmaniasis. Transbound Emerg. Dis. 69, 2320–2325. doi: 10.1111/tbed.14247 34327840

[B62] Maia da Silva F. Naiff R. D. Marcili A. Gordo M. D’Affonseca Neto J. A. Naiff M. F.. (2008). Infection rates and genotypes of *Trypanosoma rangeli* and T. cruzi infecting free-ranging *Saguinus bicolor* (Callitrichidae), a critically endangered primate of the Amazon Rainforest. Acta Trop. 107, 168–173. doi: 10.1016/j.actatropica.2008.05.015 18603222

[B63] Malta M. C. C. Tinoco H. P. Xavier M. N. Vieira A. L. S. Costa É. A. Santos R. L. (2010). Naturally acquired visceral leishmaniasis in non-human primates in Brazil. Vet. Parasitol. 169, 193–197. doi: 10.1016/j.vetpar.2009.12.016 20056328

[B64] Marcili A. Lima L. Valente V. C. Valente S. A. Batista J. S. Junqueira A. C. V.. (2009). Comparative phylogeography of *Trypanosoma cruzi* TCIIc: New hosts, association with terrestrial ecotopes, and spatial clustering. Infect. Genet. Evol. 9, 1265–1274. doi: 10.1016/j.meegid.2009.07.003 19632356

[B65] McLennan M. R. Spagnoletti N. Hockings K. J. (2017). The implications of primate behavioral flexibility for sustainable human–primate coexistence in anthropogenic habitats. Int. J. Primatol. 38, 105–121. doi: 10.1007/s10764-017-9962-0

[B66] McMurdie P. J. Holmes S. (2013). phyloseq: an R package for reproducible interactive analysis and graphics of microbiome census data. PloS One 8, e61217. doi: 10.1371/journal.pone.0061217 23630581 PMC3632530

[B67] Metzdorf I. P. da Costa Lima M. S. de Fatima Cepa Matos M. de Souza Filho A. F. de Souza Tsujisaki R. A. Franco K. G.. (2017). Molecular characterization of *Leishmania infantum* in domestic cats in a region of Brazil endemic for human and canine visceral leishmaniasis. Acta Trop. 166, 121–125. doi: 10.1016/j.actatropica.2016.11.013 27851895

[B68] Michael R. G. Joseph S. (2012). Molecular cloning: a laboratory manual. 4th ed Vol. 1 (New York: Cold Spring Harbor).

[B69] Mitchell G. C. Baker J. H. Sleigh M. A. (1988). Feeding of a freshwater flagellate, *bodo saltans*, on diverse bacteria. J. Protozool. 35, 219–222. doi: 10.1111/j.1550-7408.1988.tb04327

[B70] Naiff R. D. Barrett T. V. (2013). *Trypanosoma* (*Megatrypanum*) *lainsoni* n. sp. from *Mesomys hispidus* (Rodentia: Echimyidae) in Brazil: trypomastigotes described from experimentally infected laboratory mice. Parasite 20, 51. doi: 10.1051/parasite/2013049 24309069 PMC3853975

[B71] Nantes W. A. G. Liberal S. C. Santos F. M. Dario M. A. Mukoyama L. T. H. Woidella K. B.. (2024). Viperidae snakes infected by mammalian-associated trypanosomatids and a free-living kinetoplastid. Infect. Genet. Evol. 123, 105630. doi: 10.1016/j.meegid.2024.105630 38936526

[B72] Nantes W. A. G. Santos F. M. de Macedo G. C. Barreto W. T. G. Gonçalves L. R. Rodrigues M. S.. (2021). Trypanosomatid species in *Didelphis albiventris* from urban forest fragments. Parasitol. Res. 120, 223–231. doi: 10.1007/s00436-020-06921-y 33079269

[B73] Nasereddin A. Ereqat S. Al-Jawabreh A. Taradeh M. Abbasi I. Al-Jawabreh H.. (2022). Concurrent molecular characterization of sand flies and *Leishmania* parasites by amplicon-based next-generation sequencing. Parasit Vectors. 15, 262. doi: 10.1186/s13071-022-05388-3 35869485 PMC9308317

[B74] Neitzke-Abreu H. C. Costa G. B. da Silva M. N. Palacio E. da Silva Cardoso A. de Almeida P. S.. (2022). Geographic distribution of human leishmaniasis and phlebotomine sand flies in the State of Mato Grosso do Sul, Brazil. Parasit Vectors. 15, 227. doi: 10.1186/s13071-022-05353-0 35751099 PMC9233378

[B75] Nguyen L.-T. Schmidt H. A. von Haeseler A. Minh B. Q. (2015). IQ-TREE: A fast and effective stochastic algorithm for estimating maximum-likelihood phylogenies. Mol. Biol. Evol. 32, 268–274. doi: 10.1093/molbev/msu300 25371430 PMC4271533

[B76] Paiz L. M. Motoie G. Richini-Pereira V. B. Langoni H. Menozzi B. D. Tolezano J. E.. (2019). Antibodies and molecular detection of *Leishmania* (*Leishmania*) *infantum* in samples of free-ranging marmosets (Primates: Callitrichidae: *Callithrix* spp.) in an area of canine visceral Leishmaniasis in Southeastern Brazil. Vector Borne Zoonotic Dis. 19, 249–254. doi: 10.1089/vbz.2018.2348 30335584

[B77] Porfirio G. Santos F. M. Foster V. Nascimento L. F. Macedo G. C. Barreto W. T. G.. (2017). Terrestriality of wild *sapajus cay* (Illiger, 1815) as revealed by camera traps. Folia Primatol. 88, 1–8. doi: 10.1159/000464148 28365688

[B78] Råberg L. Graham A. L. Read A. F. (2009). Decomposing health: tolerance and resistance to parasites in animals. Philos. Trans. R Soc. Lond B Biol. Sci. 364, 37–49. doi: 10.1098/rstb.2008.0184 18926971 PMC2666700

[B79] Rambaut A. Drummond A. J. Xie D. Baele G. Suchard M. A. (2018). Posterior summarization in bayesian phylogenetics using tracer 1.7. Syst. Biol. 67, 901–904. doi: 10.1093/sysbio/syy032 29718447 PMC6101584

[B80] R Core Team (2022). R: A Language and Environment for Statistical Computing. (Viena: R Foundation for Statistical Computing).

[B81] Regasini L. O. Cotinguiba F. Passerini G. D. Bolzani V da S. Cicarelli R. M. B. Kato M. J.. (2009). Trypanocidal activity of *Piper arboreum* and *Piper tuberculatum* (Piperaceae). Rev. Bras. Farmacogn. 19, 199–203. doi: 10.1590/S0102-695X2009000200003

[B82] Rímoli J. Nantes R.d. S. Lázaro Júnior AÉLJ. (2012). Diet and activity patterns of black howler monkeys *Alouatta caraya* (Humboldt, 1812, Primates, Atelidae) in ecotone Cerrado-Pantanal in the left bank of Aquidauana River, Mato Grosso do Sul, Brazil. Oecol. Aust. 16, 933–948. doi: 10.4257/oeco.2012.1604.15

[B83] Rizzi A. Raddadi N. Sorlini C. Nordgrd L. Nielsen K. M. Daffonchio D. (2012). The stability and degradation of dietary DNA in the gastrointestinal tract of mammals: implications for horizontal gene transfer and the biosafety of GMOs. Crit. Rev. Food Sci. Nutr. 52, 142–161. doi: 10.1080/10408398.2010.49948 22059960

[B84] Rodhain J. (1941). Notes Sur *Trypanosoma minasense* Chagas. Identité spécifique du *Trypanosome* du Saimiri: *Chrysothrix sciureus* . Acta Biológica Bélgica 1, 187–192.

[B85] Rodrigues M. S. Lima L. Xavier S. C. d. C. Herrera H. M. Rocha F. L. Roque A. L. R.. (2019). Uncovering *Trypanosoma* spp. diversity of wild mammals by the use of DNA from blood clots. Int. J. Parasitol. Parasites Wildl. 8, 171–181. doi: 10.1016/j.ijppaw.2019.02.004 30847276 PMC6389730

[B86] Rodrigues M. S. Morelli K. A. Jansen A. M. (2017). Cytochrome c oxidase subunit 1 gene as a DNA barcode for discriminating *Trypanosoma cruzi* DTUs and closely related species. Parasit Vectors. 10, 488. doi: 10.1186/s13071-017-2457-1 29037251 PMC5644147

[B87] Rodrigues de Oliveira A. Pinheiro G. R. G. Tinoco H. P. Loyola M. E. Coelho C. M. Dias E. S.. (2019). Competence of non-human primates to transmit *Leishmania infantum* to the invertebrate vector Lutzomyia longipalpis. PloS Negl. Trop. Dis. 13, e0007313. doi: 10.1371/journal.pntd.0007313 30995227 PMC6488095

[B88] Roque A. L. R. Jansen A. M. (2014). Wild and synanthropic reservoirs of *Leishmania* species in the Americas. Int. J. Parasitol. Parasites Wildl. 3, 251–262. doi: 10.1016/j.ijppaw.2014.08.004 25426421 PMC4241529

[B89] Rovirosa-Hernández M. J. López-Monteon A. García-Orduña F. Torres-Montero J. Guzmán-Gómez D. Dumonteil E.. (2021). Natural infection with *Trypanosoma cruzi* in three species of non-human primates in southeastern Mexico: A contribution to reservoir knowledge. Acta Trop. 213, 105754. doi: 10.1016/j.actatropica.2020.105754 33166517

[B90] Santos F. M. Barreto W. T. G. de Macedo G. C. Barros J. H. da S. Xavier S. C. d. C. Garcia C. M.. (2019). The reservoir system for *Trypanosoma* (Kinetoplastida, Trypanosomatidae) species in large neotropical wetland. Acta Trop. 199, 105098. doi: 10.1016/j.actatropica.2019.105098 31356788

[B91] Santos R. L. de Oliveira A. R. (2020). Leishmaniasis in non-human primates: Clinical and pathological manifestations and potential as reservoirs. J. Med. Primatol. 49, 34–39. doi: 10.1111/jmp.12441 31595524

[B92] Santos F. M. Sano N. Y. Liberal S. C. Dario M. A. Nantes W. A. G. Alves F. M.. (2022). Kinetoplastid species maintained by a small mammal community in the Pantanal biome. Pathogens 11, 1205. doi: 10.3390/pathogens11101205 36297262 PMC9612235

[B93] Schubach A. Haddad F. Neto M. P. Degrave W. Pirmez C. Grimaldi G. Jr. (1998). Detection of *leishmania* DNA by polymerase chain reaction in scars of treated human patients. J. Infect. Dis. 178, 911–914. doi: 10.1086/515355 9728572

[B94] Silva T. R. M. Rios T. G. do Nascimento Ramos C. A. Scofield A. Lima T. A. R. F. Alves L. C.. (2022). Molecular characterization of *Trypanosoma cruzi* DTUs of the triatomine species in a Chagas disease endemic area. J. Parasit Dis. 46, 64–71. doi: 10.1007/s12639-021-01418-6 35299926 PMC8901897

[B95] Silveira F. T. Lainson R. Corbett C. E. (2004). Clinical and immunopathological spectrum of American cutaneous leishmaniasis with special reference to the disease in Amazonian Brazil: a review. Mem. Inst Oswaldo Cruz. 99, 239–251. doi: 10.1590/S0074-02762004000300001 15273794

[B96] Smith A. Clark P. Averis S. Lymbery A. J. Wayne A. F. Morris K. D.. (2008). Trypanosomes in a declining species of threatened Australian marsupial, the brush-tailed bettong *Bettongia penicillata* (Marsupialia: Potoroidae). Parasitology 135, 1329–1335. doi: 10.1017/S0031182008004824 18752704

[B97] Sousa O. E. Dawson G. A. (1976). *Trypanosome* infections in the marmoset (*Saguinus geoffroyi*) from the Panama canal zone. Am. J. Trop. Med. Hyg. 25, 407–409. doi: 10.4269/ajtmh.1976.25.407 820209

[B98] Spisák S. Solymosi N. Ittzés P. Bodor A. Kondor D. Vattay G.. (2013). Complete genes may pass from food to human blood. PloS One 8, e69805. doi: 10.1371/journal.pone.0069805 23936105 PMC3728338

[B99] Stevens J. R. (2008). Kinetoplastid phylogenetics, with special reference to the evolution of parasitic trypanosomes. Parasite 15, 226–232. doi: 10.1051/parasite/2008153226 18814685

[B100] Torres J. M. de Oliveira C. E. Santos F. M. Sano N. Y. Martinez É. V. Alves F. M.. (2024). Trypanosomatid diversity in a bat community of an urban area in Campo Grande, Mato Grosso do Sul, Brazil. Infect. Genet. Evol. 118, 105563. doi: 10.1016/j.meegid.2024.105563 38301855

[B101] Trüeb I. Portela R. D. Franke C. R. Carneiro I. O. Ribeiro G. J. Soares R. P.. (2018). *Trypanosoma cruzi* and *Leishmania* sp. Infection in wildlife from urban rainforest fragments in northeast Brazil. J. Wildl Dis. 54, 76–84. doi: 10.7589/2017-01-017 28977769

[B102] Verderane M. P. Falótico T. Resende B. D. Labruna M. B. Izar P. Ottoni E. B. (2007). Anting in a semifree-ranging group of cebus apella. Int. J. Primatol 28, 47–53. doi: 10.1007/s10764-006-9102-8

[B103] Villamizar L. H. Cardoso M.d. G. de Andrade J. Teixeira M. L. Soares M. J. (2017). Linalool, a *Piper aduncum* essential oil component, has selective activity against *Trypanosoma cruzi* trypomastigote forms at 4°C. Mem. Inst Oswaldo Cruz. 112, 131–139. doi: 10.1590/0074-02760160361 28177047 PMC5293122

[B104] Williamson R. E. Webb S. E. Dubreuil C. Lopez R. Cheves Hernandez S. Fedigan L. M.. (2021). Sharing spaces: niche differentiation in diet and substrate use among wild capuchin monkeys. Anim. Behav. 179, 317–338. doi: 10.1016/j.anbehav.2021.06.002

[B105] Zhang D. Gao F. Jakovlić I. Zou H. Zhang J. Li W. X.. (2020). PhyloSuite: An integrated and scalable desktop platform for streamlined molecular sequence data management and evolutionary phylogenetics studies. Mol. Ecol. Resour. 20, 348–355. doi: 10.1111/1755-0998.13096 31599058

[B106] Ziccardi M. Lourenço-de-Oliveira R. Lainson R. Brígido M.do C.d. Muniz J. A. P. C. (2000). Trypanosomes of non-human primates from the National Centre of Primates, Ananindeua, State of Pará, Brazil. Mem. Inst Oswaldo Cruz. 95, 157–159. doi: 10.1590/S0074-0276200000020000 10733732

[B107] Zingales B. Bartholomeu D. C. (2022). *Trypanosoma cruzi* genetic diversity: impact on transmission cycles and Chagas disease. Mem. Inst Oswaldo Cruz. 117, e210193. doi: 10.1590/0074-02760210193 35544857 PMC9088421

