# Supplementary material for: Non-human primates as indicators of Kinetoplastida diversity in an urban environment in Midwest Brazil
Source: Front Parasitol. 2025 Feb 17;4:1547701. doi: 10.3389/fpara.2025.1547701 (PMC11873808; doi:10.3389/fpara.2025.1547701)
Supplement: Supplementary file 1 [file Table1.docx]

Table S1: Sequencing data quality for the analyzed samples expressed by: input—number of reads in raw fastq files; filtered—number of reads after preliminary quality filtering; denoised (F/R)—number of reads after quality filtering; merged— number of merged forward-reverse reads; nonchim—number of merged reads after removal of chimera sequences; % passed—relative number of passed reads after all the above steps.

| **N** | **UCDB** | **Host** | **Template** | **SPP** | **Sex** | **Area** | **input** | **filtered** | **denoisedF** | **denoisedR** | **merged** | **nonchim** | **%** |
| --- | --- | --- | --- | --- | --- | --- | --- | --- | --- | --- | --- | --- | --- |
| 1 | 297 | A1 | Clots | *Sapajus cay* | Male | ISV | 115767 | 87793 | 87138 | 86950 | 82711 | 81034 | 97.97% |
| 2 | 298 | A2 | Clots | *Sapajus cay* | Female | ISV | 115602 | 84752 | 84219 | 84136 | 81098 | 79235 | 97.70% |
| 3 | 304 | A3 | Clots | *Sapajus cay* | Female | PEMS | 105234 | 85365 | 85013 | 84955 | 82653 | 80975 | 97.97% |
| 4 | 306 | A4 | Clots | *Sapajus cay* | Female | PEMS | 117398 | 96500 | 95569 | 96043 | 90482 | 88555 | 97.87% |
| 5 | 460 | A5 | Clots | *Sapajus cay* | Female | ISV | 100897 | 73884 | 73454 | 73551 | 70268 | 70046 | 99.68% |
| 6 | 480B | A6 | Clots + Blood | *Sapajus cay* | Female | PEMS | 119507 | 98190 | 97768 | 97889 | 95632 | 88173 | 92.20% |
| 7 | 300B | A7 | Clots + Blood | *Sapajus cay* | Male | PEMS | 105136 | 84678 | 84240 | 84357 | 80812 | 78893 | 97.63% |
| 8 | 302 | A8 | Clots + Blood | *Sapajus cay* | Male | PEMS | 103875 | 71528 | 71158 | 70927 | 66430 | 66164 | 99.60% |
| 9 | 489 | A9 | Clots + Blood | *Sapajus cay* | Male | ISV | 106670 | 88626 | 88462 | 88307 | 85353 | 80995 | 94.89% |
| 10 | 496 | A10 | Clots + Blood | *Sapajus cay* | Male | CRAS | 100381 | 75499 | 74733 | 75243 | 71774 | 70956 | 98.86% |
| 11 | 548 | A11 | Clots + Blood | *Sapajus cay* | Male | CRAS | 112899 | 91477 | 91301 | 90767 | 88960 | 86834 | 97.61% |
| 12 | 549 | A12 | Clots + Blood | *Sapajus cay* | Male | CRAS | 110757 | 90114 | 89686 | 89582 | 85818 | 82002 | 95.55% |
| 13 | 307 | A13 | Blood | *Sapajus cay* | Male | PEMS | 101237 | 82995 | 82130 | 82187 | 76729 | 75739 | 98.71% |
| 14 | 457 | A14 | Blood | *Sapajus cay* | Male | PEMS | 103479 | 84890 | 83874 | 83866 | 78891 | 77386 | 98.09% |
| 15 | 458 | A15 | Blood | *Sapajus cay* | Female | PEMS | 101256 | 78174 | 76134 | 76264 | 67514 | 63693 | 94.34% |
| 16 | 486 | A16 | Blood | *Sapajus cay* | Female | PEMS | 100908 | 82926 | 82046 | 82340 | 76826 | 75192 | 97.87% |
| 17 | 488 | A17 | Blood | *Sapajus cay* | Male | PEMS | 112866 | 89678 | 88626 | 88618 | 83012 | 81767 | 98.50% |
| 18 | 490 | A18 | Blood | *Sapajus cay* | Female | PEMS | 111683 | 80908 | 79887 | 79970 | 72445 | 64645 | 89.23% |
| 19 | 304B | A19 | Blood | *Sapajus cay* | Female | PEMS | 112027 | 68742 | 67376 | 67414 | 60777 | 58950 | 96.99% |
| 20 | 491 | A20 | Blood | *Sapajus cay* | Female | PEMS | 105576 | 83069 | 81898 | 81672 | 72919 | 68121 | 93.42% |
| 21 | 492 | A21 | Blood | *Sapajus cay* | Female | PEMS | 119896 | 87649 | 85708 | 85461 | 74698 | 69259 | 92.72% |
| 22 | 493 | A22 | Blood | *Sapajus cay* | Female | CRAS | 39845 | 30698 | 30084 | 29957 | 27605 | 26346 | 95.44% |
| 23 | 494 | A23 | Blood | *Sapajus cay* | Male | CRAS | 106489 | 66557 | 65262 | 65181 | 59194 | 58005 | 97.99% |
| 24 | 550 | A24 | Blood | *Sapajus cay* | Male | CRAS | 118833 | 66928 | 66223 | 66209 | 62966 | 61171 | 97.15% |
| 25 | 551 | A25 | Blood | *Sapajus cay* | Male | CRAS | 50863 | 42287 | 41855 | 41876 | 39957 | 38013 | 95.13% |
| 26 | 552 | A26 | Blood | *Alouatta caraya* | Female | CRAS | 117688 | 82847 | 81978 | 81907 | 72235 | 63982 | 88.57% |
| 27 | 553 | A27 | Blood | *Alouatta caraya* | Female | CRAS | 115801 | 89323 | 87609 | 87442 | 74797 | 69381 | 92.76% |
| 28 | 554 | A28 | Blood | *Alouatta caraya* | Male | CRAS | 104424 | 83377 | 83110 | 82824 | 80276 | 78583 | 97.89% |
| 29 | 555 | A29 | Blood | *Alouatta caraya* | Female | CRAS | 102062 | 85981 | 85182 | 85116 | 82089 | 80870 | 98.52% |
| 30 | 465 | A30 | Bone marrow | *Sapajus cay* | Male | PEMS | 105675 | 84855 | 83811 | 83592 | 77785 | 76044 | 97.76% |
| 31 | 466 | A31 | Bone marrow | *Sapajus cay* | Female | PEMS | 101341 | 81362 | 79892 | 80025 | 73886 | 73220 | 99.10% |
| 32 | 495 | A32 | Bone marrow | *Sapajus cay* | Male | CRAS | 84112 | 66437 | 65011 | 64669 | 56578 | 53442 | 94.46% |
